# Supplementary material for: The landscape of human mutually exclusive splicing
Source: Mol Syst Biol. 2017 Dec 14;13(12):959. doi: 10.15252/msb.20177728 (PMC5740500; doi:10.15252/msb.20177728)
Supplement: Supplementary file 1 — Appendix [file MSB-13-959-s001.pdf]

Appendix

The landscape of human mutually exclusive splicing

Klas Hatje[1,2,#], Raza-Ur Rahman[2,3], Ramon O. Vidal[2,%], Dominic Simm[1,4], Björn Hammesfahr[1,$], Vikas Bansal[2,3], Ashish Rajput[2,3], Michel Edwar Mickael[2,3], Ting Sun[2,3], Stefan Bonn[2,3,5,§] & Martin Kollmar[1,§]

[1] Group Systems Biology of Motor Proteins, Department of NMR-based Structural Biology, Max-Planck-Institute for Biophysical Chemistry, Göttingen, Germany

[2] Group of Computational Systems Biology, German Center for Neurodegenerative Diseases, Göttingen, Germany

[3] Institute of Medical Systems Biology, Center for Molecular Neurobiology, University Clinic Hamburg-Eppendorf, Hamburg, Germany

[4] Theoretical Computer Science and Algorithmic Methods, Institute of Computer Science, Georg-August-University Göttingen, Germany

[5] German Center for Neurodegenerative Diseases, Tübingen, Germany

# Current address: Roche Pharmaceutical Research and Early Development, Pharmaceutical Sciences, Roche Innovation Center Basel, F. Hoffmann-La Roche Ltd., Basel, Switzerland

% Current address: Max-Delbrück-Center for Molecular Medicine, Berlin, Germany

$ Current address: Research and Development - Data Management (RD-DM), KWS SAAT SE, Einbeck, Germany

§ Corresponding authors.

Table of contents

Appendix References 1
Appendix Figures 2
Appendix Fig. S1: MXE candidate annotation. 2
Appendix Fig. S2: Schematic representation of the MXE candidate categories and examples from the human genome annotation.. 4
Appendix Fig. S3: RNA-seq coverage of the MXE candidates. 6
Appendix Fig. S4: Types of terminal mutually exclusive exon candidates. 7
Appendix Fig. S5: MXE candidates validated by increasingly stringent criteria. 8
Appendix Fig. S6: Example for MXEs in direct neighbourhood to other splice variants. 9
Appendix Fig. S7: Number of criteria defining clusters of three and five MXEs, respectively. 10
Appendix Fig. S8: RNA-Seq mapping of the mutually exclusive exome. 11
Appendix Fig. S9: Coverage of the mutually exclusive exome in different RNA-seq projects. 12
Appendix Fig. S10: RNA-Seq mapping of the mutually exclusive exome. 13
Appendix Fig. S11: Validation of MXEs by using increasing amounts of RNA-seq data. 15
Appendix Fig. S12: GTEX validation of MXEs with two annotated exons. 16
Appendix Fig. S13: qPCR validation of MXEs. 17
Appendix Fig. S14: MXE and cassette exon GO enrichment. 18
Appendix Fig. S15: Human genes with clusters of multiple MXEs. 19
Appendix Fig. S16: Annotated *CUX1* isoforms with those exons highlighted that were validated as MXEs. 20
Appendix Fig. S17: MXE splicing of *CUX1* 21
Appendix Fig. S18: U2/U12 incompatibility. 22
Appendix Fig. S19: Length distribution of the introns between MXEs. 24
Appendix Fig. S20: Analysis of MXE reading frame. 25
Appendix Fig. S21: Example highlighting several aspects of generating the MXE candidate list, MXE validation, and splicing mechanism. 27
Appendix Fig. S22: Protein structure analysis of the MXE-encoded regions. 28
Appendix Fig. S23: Annotated and novel MXE cluster expression. 31
Appendix Fig. S24: Percentage of differentially expressed MXEs per cluster. 32
Appendix Fig. S25: MXE expression-specificity. 33
Appendix Fig. S26A: The Solute Carrier Family 8 Member A1 gene (*SLC8A1*) is an example of an MXE cluster with a large differences between the Gini-values of the MXEs. 34
Appendix Fig. S26B: The Synaptosome Associated Protein 25 (*SNAP25*) is an example of an MXE cluster with a small differences between the Gini-values of both MXEs. 35
Appendix Fig. S27: Network plot of diseases, genes, and protein classes. 36
Appendix Fig. S28: Differential expression of and pathogenic mutations in MXEs of *ACTN4*. 37
Appendix Fig. S29: Comparison of the expression level and tissue distribution of known exons and their corresponding novel partner MXEs. 39
Appendix Fig. S30: *TPM1* MXE expression and splicing. 41
Appendix Fig. S31: *SLC25A3* MXE Expression. 42
Appendix Fig. S32: *FAR1* MXE expression and splicing. 43

Appendix Fig. S33: Gain and loss of clusters of MXEs. 44
Appendix Fig. S34: Orthologous human and *Drosophila* genes containing MXEs. 45
Appendix Fig. S35: Orthologous human and *Drosophila* genes containing MXEs. 48
Appendix Fig. S36: Examples of orthologous human and *Drosophila* genes containing MXEs. 50

Appendix References

Hedges SB, Dudley J, Kumar S. 2006. TimeTree: a public knowledge-base of divergence times among organisms. *Bioinforma Oxf Engl* **22**: 2971–2972.

Kaplan JM, Kim SH, North KN, Rennke H, Correia LA, Tong HQ, Mathis BJ, Rodríguez-Pérez JC, Allen PG, Beggs AH, et al. 2000. Mutations in ACTN4, encoding alpha-actinin-4, cause familial focal segmental glomerulosclerosis. *Nat Genet* **24**: 251–256.

Lek M, MacArthur DG, Yang N, North KN. 2010. Phylogenetic analysis of gene structure and alternative splicing in alpha-actinins. *Mol Biol Evol* **27**: 773–780.

Magen A, Ast G. 2005. The importance of being divisible by three in alternative splicing. *Nucleic Acids Res* **33**: 5574–5582.

Tang ZZ, Sharma S, Zheng S, Chawla G, Nikolic J, Black DL. 2011. Regulation of the mutually exclusive exons 8a and 8 in the CaV1.2 calcium channel transcript by polypyrimidine tract-binding protein. *J Biol Chem* **286**: 10007–10016.

Appendix Figures

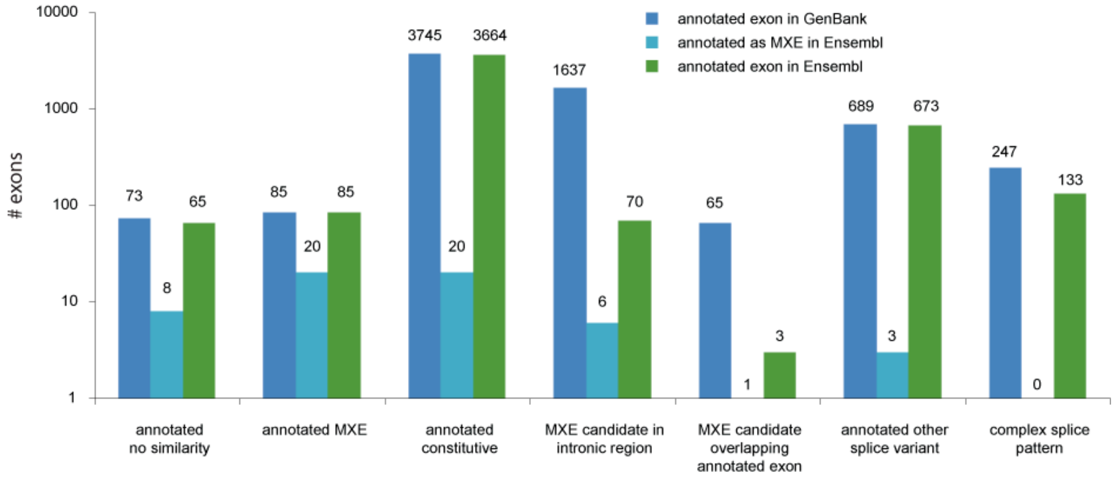

Appendix Fig. S1: MXE candidate annotation. Comparison of GenBank and Ensembl annotations. Because the generation of the MXE candidate list depends on the underlying set of exon annotations, we used the GenBank exon annotation as reference and looked how these MXE candidates are annotated in the latest Ensembl (release 37.75) annotation. Overall, there is low concordance in the annotation of exons as mutually exclusive. For example, only 18% of the 'annotated MXEs' in GenBank are also annotated as MXE in Ensembl, while 95% of these exons are present in the annotation. 20 exons annotated as constitutive in GenBank are annotated as MXEs in Ensembl. 70 of the 1637 exons not present in GenBank, which we identified in our search for MXE candidates in intronic regions ('novel exons'), are present in the Ensembl annotation, though not annotated as MXEs, and six of these 1637 exons are even annotated as MXEs.

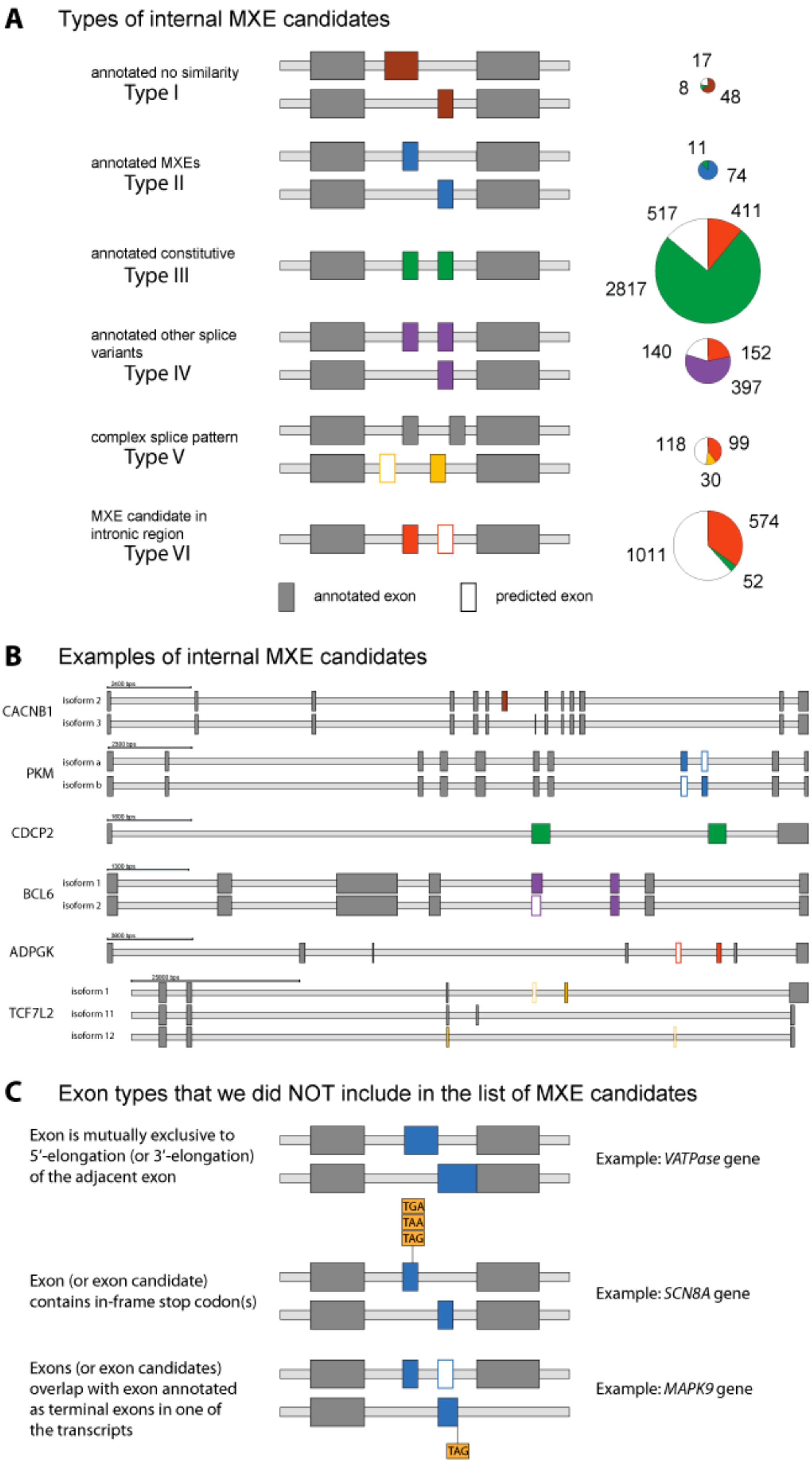
A
Types of internal MXE candidates
annotated no similarity
Type I
17
8
48
annotated MXEs
Type II
11
74
annotated constitutive
Type III
517
411
2817
annotated other splice variants
Type IV
140
152
397
complex splice pattern
Type V
118
99
30
MXE candidate in intronic region
Type VI
574
1011
52
annotated exon
predicted exon
B
Examples of internal MXE candidates
CACNB1
isoform 2
isoform 3
PKM
isoform a
isoform b
CDCP2
BCL6
isoform 1
isoform 2
ADPGK
TCF7L2
isoform 1
isoform 11
isoform 12
C
Exon types that we did NOT include in the list of MXE candidates
Exon is mutually exclusive to 5′-elongation (or 3′-elongation) of the adjacent exon
Example: VATPase gene
TGA
TAA
TAG
Exon (or exon candidate) contains in-frame stop codon(s)
Example: SCN8A gene
Exons (or exon candidates) overlap with exon annotated as terminal exons in one of the transcripts
Example: MAPK9 gene
TAG

Appendix Fig. S2: Schematic representation of the MXE candidate categories and examples from the human genome annotation. A) Because of the preliminary character of the genome annotation (19201 protein coding genes) we discriminated six types of exons for the analysis. 82 genes contained 87 annotated clusters of MXEs of which 85 MXEs had similar length and sequence (Type II) while the other annotated MXEs did not show any similarity (Type I). 1545 genes contained constitutive and differentially included exons (3745 and 689 exons, respectively) with characteristics of MXEs (Type III and Type IV, respectively). In 1014 genes we predict 1637 new MXE candidates in intronic regions (Type VI). 247 new MXE candidates were predicted in 115 genes within regions showing complex splice patterns (Type V). The pie charts show the number of MXE candidates (represented by the size of the pie) divided into i) the number of exons validated as the respective type (in the colour of the type), ii) the number of exons validated as MXEs (in red) or constitutive (in green), and iii) the number of MXE candidates, for which splice junction reads were missing thus not allowing their annotation (in white). B) Examples for the various types of annotations using the same colour scheme for the MXE candidates as in the schematic representation. C) There are mainly three types of alternatively spliced exons that we did not include in the MXE candidate list, exons that are mutually exclusive to exonic region otherwise part of another exon, exons including in-frame stop codons, and exons mutually exclusive to terminal exons. Alternative terminal exons were ignored because they are mutually included not through alternative splicing but alternative promoter usage, and alternative cleavage and polyadenylation (Appendix Fig. S4).

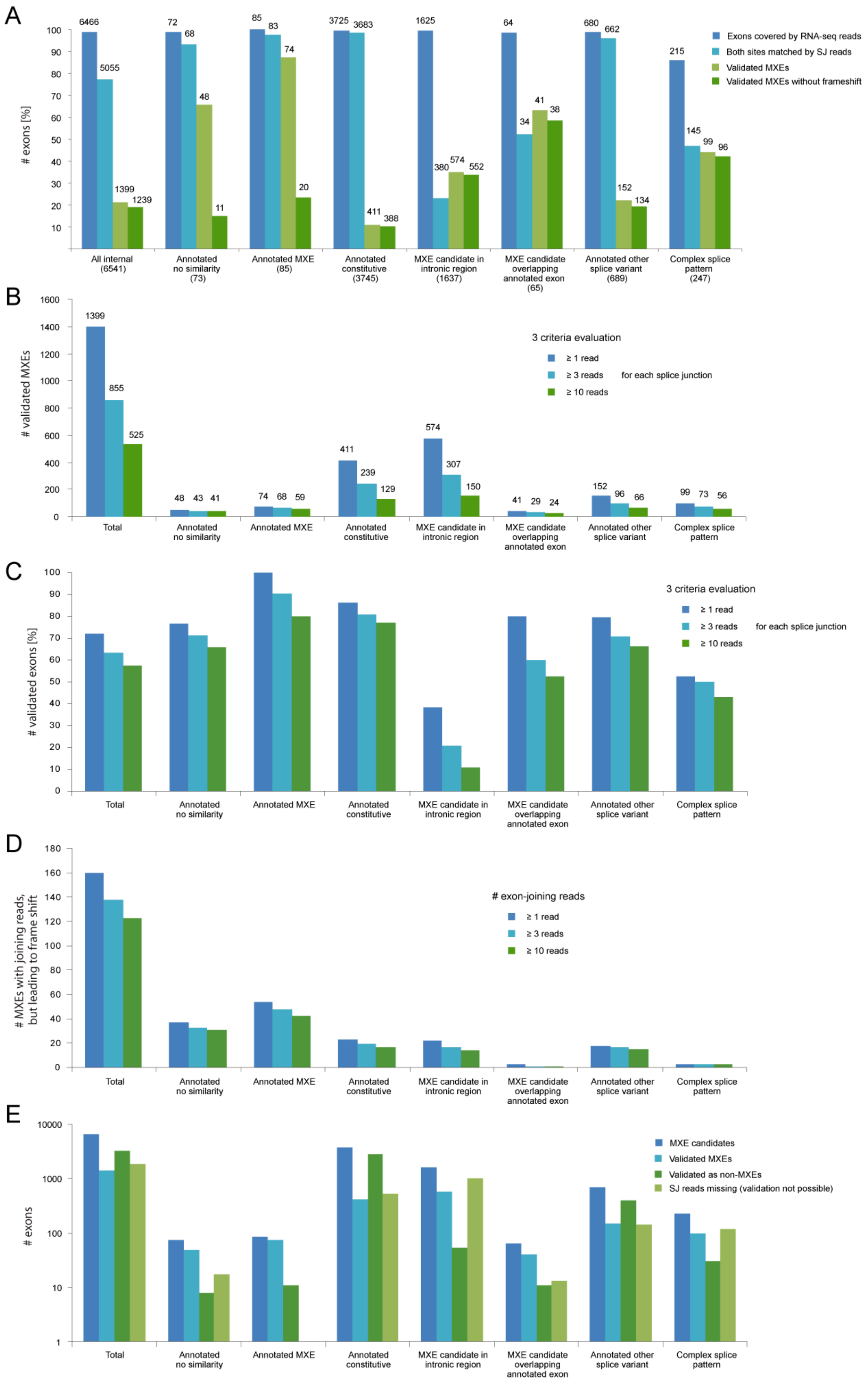

A
exons [%]
6466
5055
1399
1239
72
68
48
11
85
83
74
20
3725
3683
411
388
1625
380
574
552
64
34
41
38
680
662
152
134
215
145
99
96
Exons covered by RNA-seq reads
Both sites matched by SJ reads
Validated MXEs
Validated MXEs without frameshift
All internal (6541)
Annotated no similarity (73)
Annotated MXE (85)
Annotated constitutive (3745)
MXE candidate in intronic region (1637)
MXE candidate overlapping annotated exon (65)
Annotated other splice variant (689)
Complex splice pattern (247)
B
validated MXEs
1399
855
525
48
43
41
74
68
59
411
239
129
574
307
150
41
29
24
152
96
66
99
73
56
3 criteria evaluation
≥ 1 read
≥ 3 reads
≥ 10 reads
for each splice junction
Total
Annotated no similarity
Annotated MXE
Annotated constitutive
MXE candidate in intronic region
MXE candidate overlapping annotated exon
Annotated other splice variant
Complex splice pattern
C
validated exons [%]
3 criteria evaluation
≥ 1 read
≥ 3 reads
≥ 10 reads
for each splice junction
D
MXEs with joining reads, but leading to frame shift
exon-joining reads
≥ 1 read
≥ 3 reads
≥ 10 reads
E
exons
MXE candidates
Validated MXEs
Validated as non-MXEs
SJ reads missing (validation not possible)

Appendix Fig. S3: RNA-seq coverage of the MXE candidates. A) The barplot shows the percentage of MXE candidates for which mapping RNA-seq reads have been found (dark blue) and of which both exon borders could be validated by SJ reads (light blue). For comparison, the percentage of validated MXEs is shown with (light green) and without (dark green) presence of MXE-joining reads, which would, however, lead to a frame-shift and nonsense mediated decay of the mRNA. B) Dependency of the number of validated MXEs, sorted by MXE candidate type, on increasing number of SJ read support. SJ read support means, that all respective splice junctions must be supported by the respective numbers of SJ reads. C) In this plot we wanted to analyse the "missing SJ read data". If we require more SJ read data to support the decision how many exons can still unambiguously be classified as non-MXE or MXE? Thus, this plot shows the percentage of exons that could be validated as non-MXE or MXE depending on ≥1, ≥3 or ≥10 reads supporting splice junctions and combined inclusion (in case of non-MXEs). D) Number of MXEs with exon-joining reads, that would, however, lead to a frame-shift and potential premature termination in case of combined inclusion in the transcript. This criterion applies to many of the annotated MXEs and other annotated exons, but only to a few of the MXEs newly predicted in introns. E) The barplot contrasts validated MXEs with MXE candidates validated as being constitutively or differentially included spliced. In addition, the numbers of MXE candidates are given, for which data are not available.

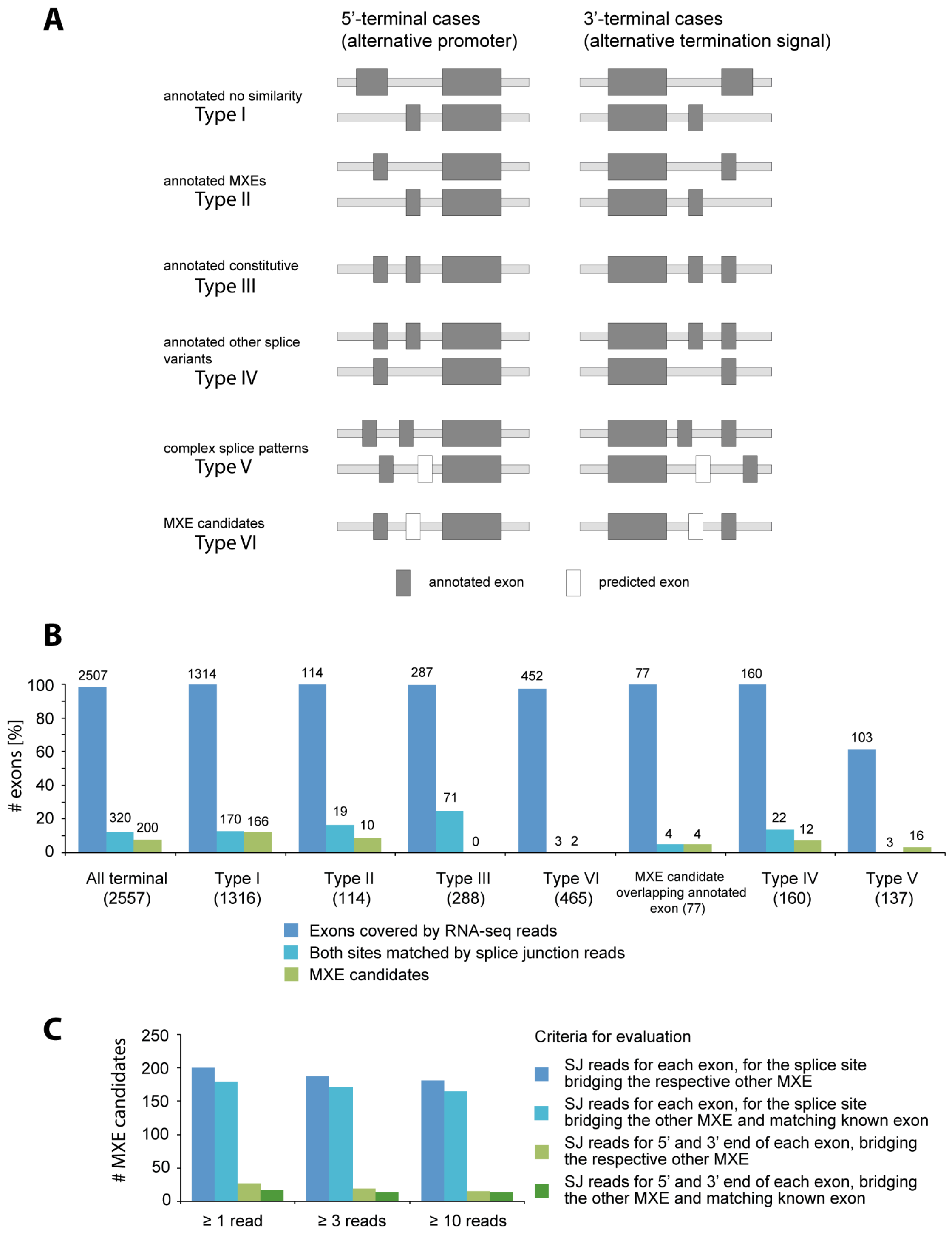

Appendix Fig. S4: Types of terminal mutually exclusive exon candidates. A) Terminal mutually exclusive exon candidates might become MXEs if further upstream or downstream exons (non-coding or coding) will be identified. The same types were distinguished as for the internal MXE candidates. B) 48% (1193 of 2507) of the mutually exclusive terminal exons (annotated and predicted) have similar lengths and sequences making them the most likely candidates for real MXEs. C) 200 of these could be validated to be spliced in a mutually exclusive manner, with splice-junction reads mapping up- (in case of the 5' terminal cluster) or downstream (in case of the 3' terminal cluster) of the cluster of MXE candidates.

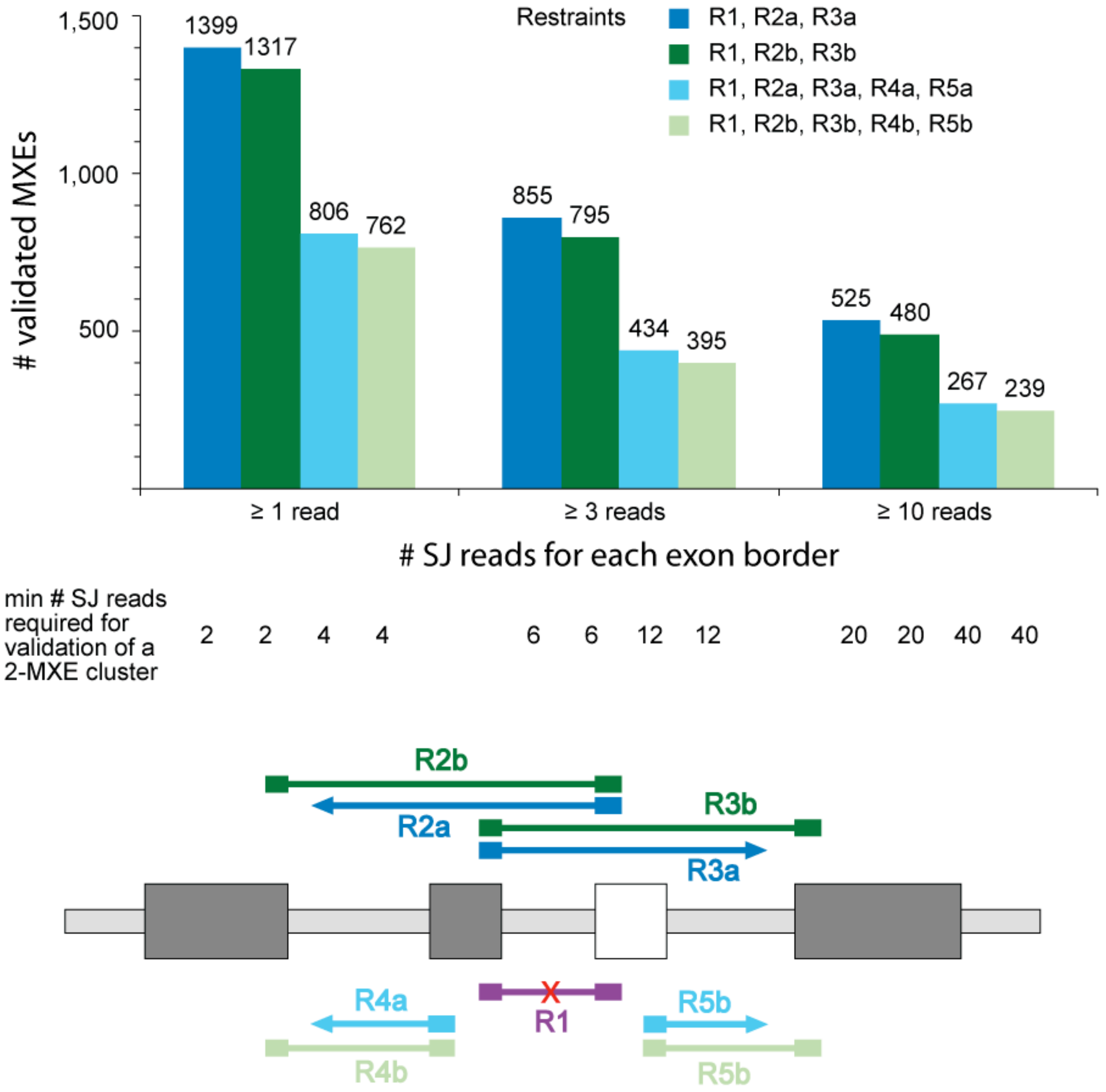

Appendix Fig. S5: MXE candidates validated by increasingly stringent criteria. The minimum requirement for defining a pair of MXEs is three constraints. There cannot exist any read mapping from one to another MXE, except for those leading to a frame shift (crossed purple splice-junction read). Without this constraint, MXEs cannot be distinguished from neighbouring differentially included exons. There must be splice-junction reads for each MXE bridging the respective other MXE and matching genomic region up- or downstream of the respective other MXE (dark blue restraints, type "a"). Under more stringent conditions, also splice-junction reads for the other exon border are required resulting in five criteria (light blue restraints). The criterion, that MXE bridging reads must only map to somewhere up- or downstream, respectively, of the cluster (indicated by arrows), takes into account that the annotated exons neighbouring the clusters of MXEs might not themselves be constitutive but alternative exons like in the *NCX1* gene (Appendix Fig. S6). In most cases, the exons neighbouring the clusters of MXEs are constitutive, which is shown by the number of validated MXEs if splice-junction reads must map to annotated neighbouring exons (compare dark and light green bars to dark and light blue bars, respectively). R = restraint.

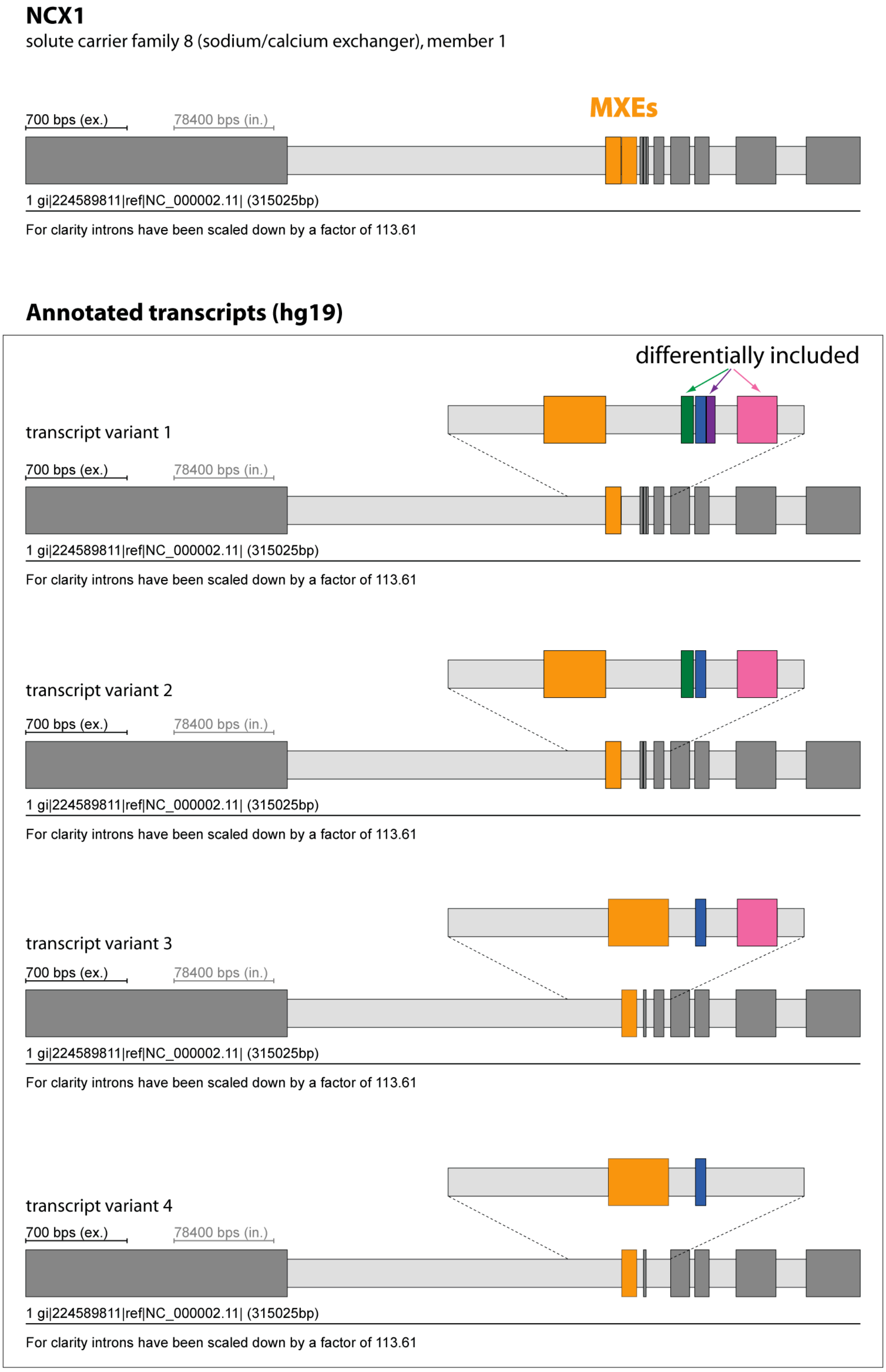

Appendix Fig. S6: Example for MXEs in direct neighbourhood to other splice variants. In the *NCX1* gene, the cluster of MXEs is adjacent to four differentially included exons. Both MXEs are annotated, and zoomed views of the respective genomic region are shown to highlight the various annotations. All transcripts are represented 5' to 3'. Constitutive exons are shown as dark grey bars, mutually exclusive exons are coloured in orange, and differentially included exons in green, blue, purple and magenta. Same alternatively spliced exons are shown in same colour across the transcript isoforms.

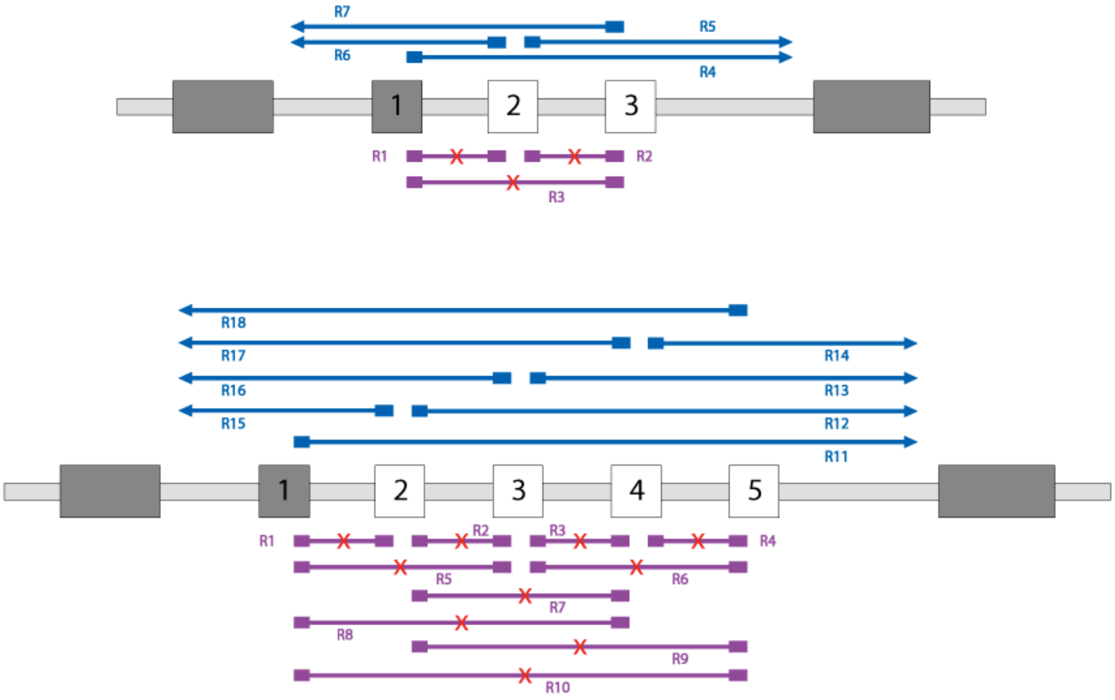

Appendix Fig. S7: Number of criteria defining clusters of three and five MXEs, respectively. The validation of larger clusters of MXEs requires an exponentially increasing number of constraints. As example, the minimum criteria for defining sets of three and five MXEs, respectively, are shown. Crossed purple splice-junction reads symbolize reads mapping from one to another MXE that cannot exist except for those leading to a frame shift. Dark blue reads illustrate splice-junction reads for each MXE bridging all other MXEs of the cluster and matching genomic region up- or downstream of the respective other MXEs. R = restraint.

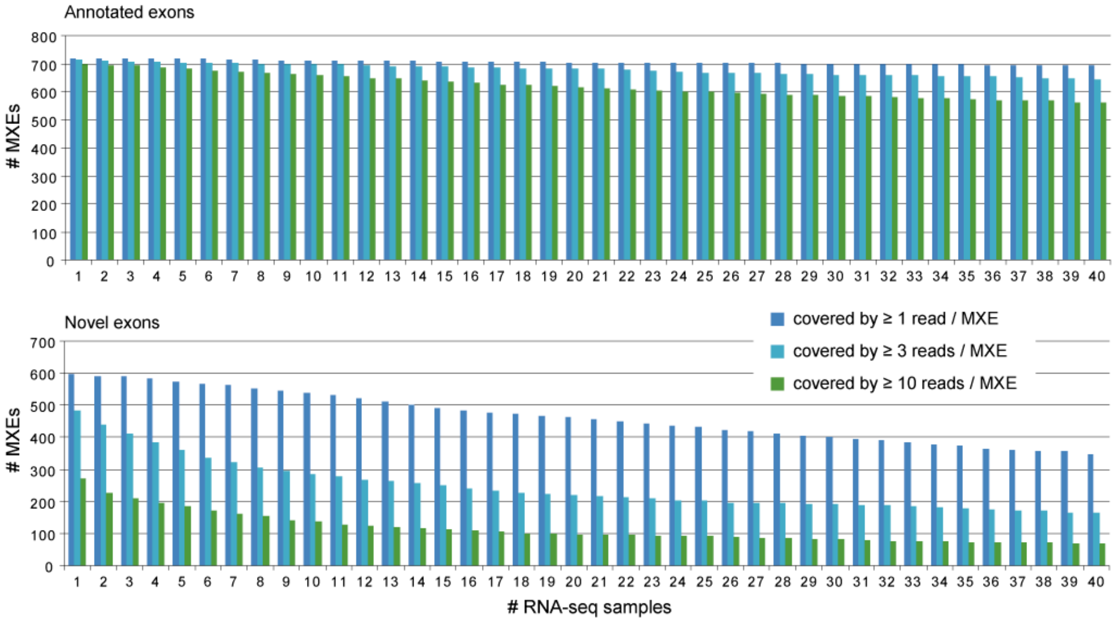

Appendix Fig. S8: RNA-Seq mapping of the mutually exclusive exome. Although the overall transcript and splice-junction support for all 1399 MXEs is relatively high, there are strong differences between the mappings of the annotated "known" exons of the MXEs compared to the MXEs predicted in intronic regions. The diagram displays number of already annotated exons (upper plot) and exons newly predicted within introns (lower plot) found in at least the number of given datasets (excluding the 16 paired-end read datasets from the Illumina Body Map project for this analysis). The 54 predicted exons overlapping with exons in other transcript isoforms were omitted in this analysis due to read mapping ambiguities. Support for 94 % of the already annotated exons is found in at least 40 datasets, whereas RNA-Seq data mapping to 74 % of the newly predicted exons are found in at least 5 datasets and 24 % in at least 40 datasets.

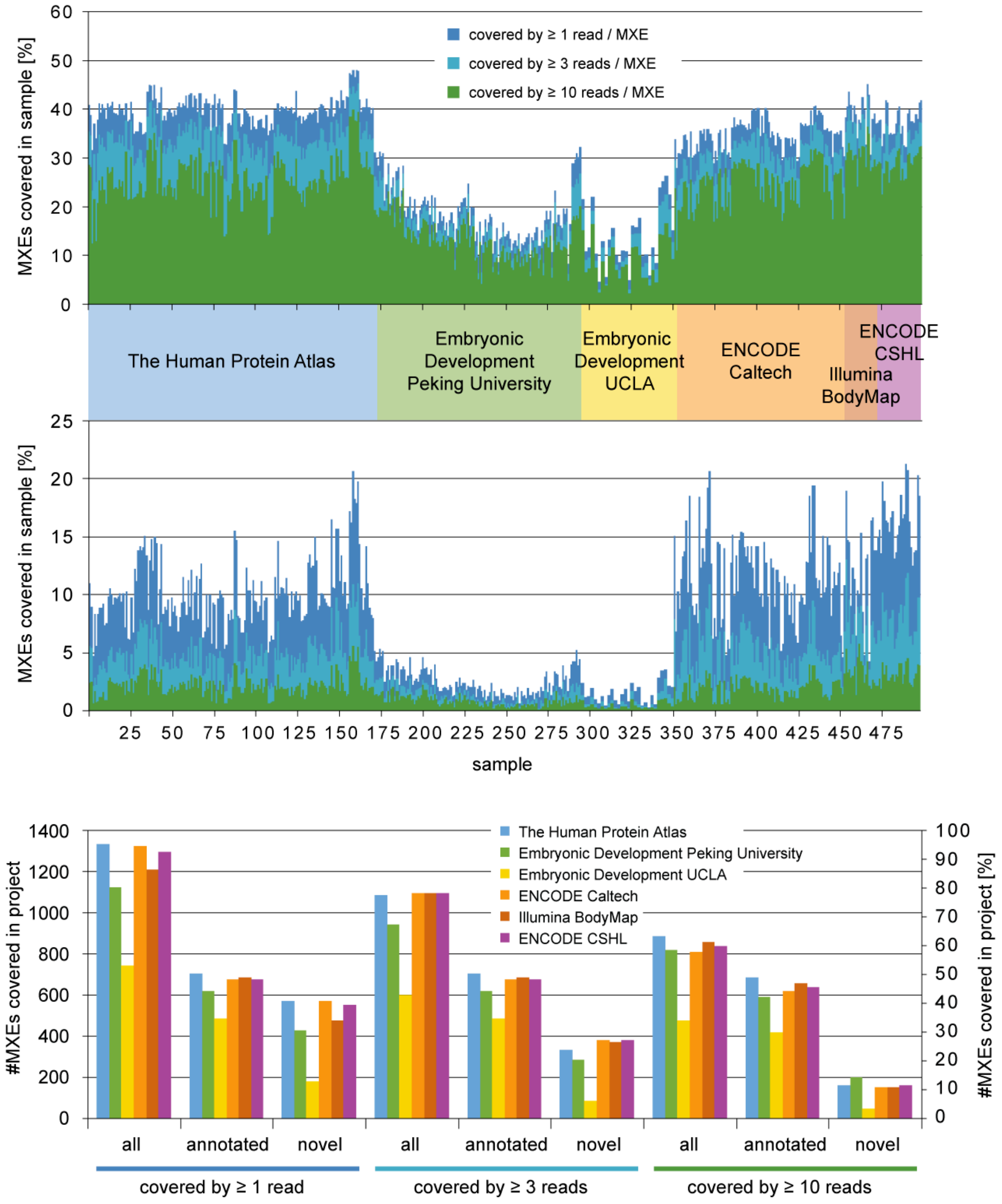

Appendix Fig. S9: Coverage of the mutually exclusive exome in different RNA-seq projects. The plots show the number of annotated exons (upper plot) versus the number of novel exons (lower plot) for each of 499 analysed RNA-seq samples (excluding the 16 paired-end read datasets from the Illumina Body Map project in this analysis). The comparison shows that the novel exons are in general lower expressed than the annotated exons, but also suggests that the novel exons are expressed more tissue and developmental stage specific.

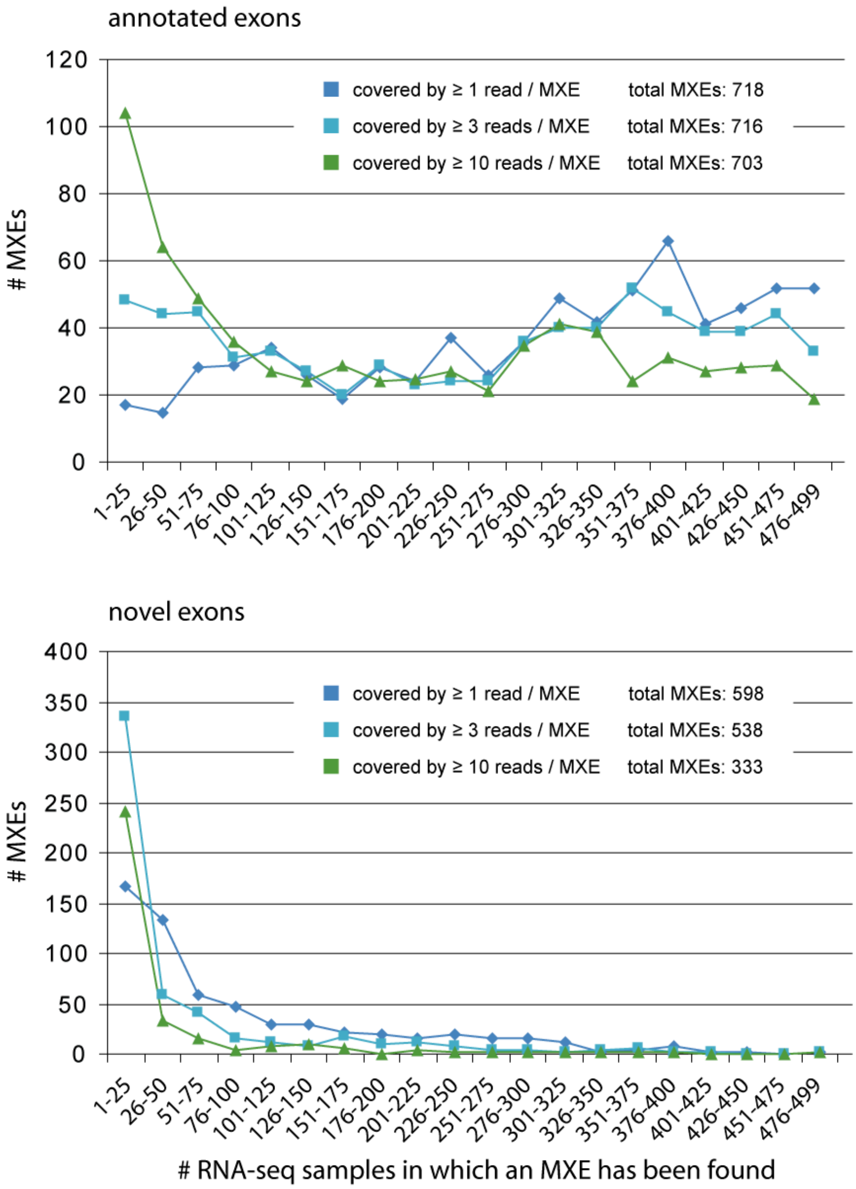

Appendix Fig. S10: RNA-Seq mapping of the mutually exclusive exome. While 65% of the annotated exons are present in more than half of the 499 RNA-Seq datasets, almost all newly predicted MXEs are present in less then 100 of the datasets.

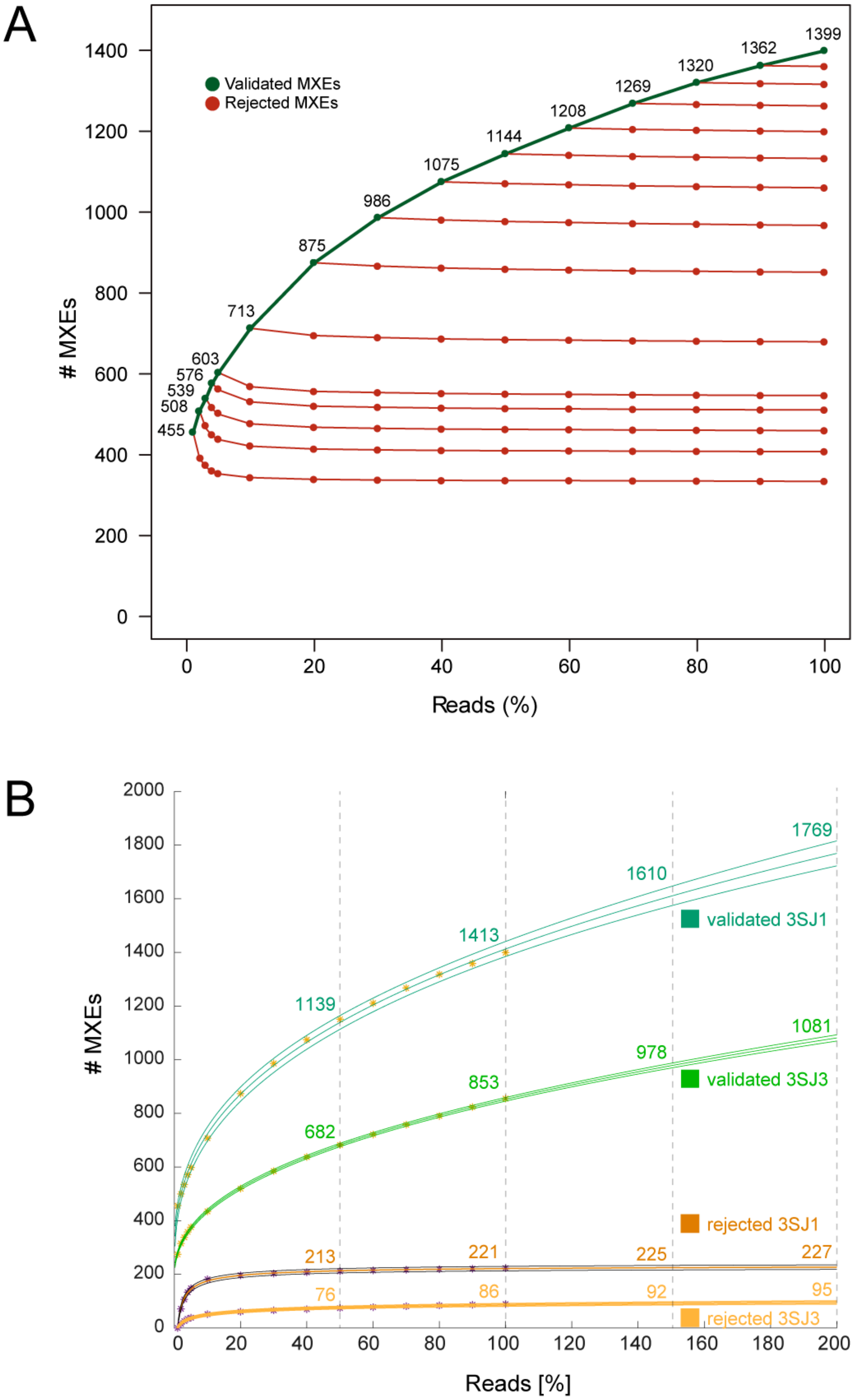
A
Validated MXEs
Rejected MXEs
MXEs
Reads (%)
455
508
539
576
603
713
875
986
1075
1144
1208
1269
1320
1362
1399
B
MXEs
Reads [%]
validated 3SJ1
validated 3SJ3
rejected 3SJ1
rejected 3SJ3
1139
1413
1610
1769
682
853
978
1081
213
221
225
227
76
86
92
95

Appendix Fig. S11: Validation of MXEs by using increasing amounts of RNA-seq data. A) The figure shows the increase of the validated MXEs (green) and the saturation of the false positives MXEs (red) in dependence of different percentages of the total RNA-seq data used. The rejection of MXE candidates almost saturates at 20% of the RNA-seq data. MXEs were verified in each experiment using the same criteria as for the analysis using all RNA-seq data (Appendix Fig. S5), namely the presence of SJ reads bridging the respective other MXE candidates of the cluster and the absence of reads that would join MXE candidates of the cluster. B) To estimate the potential increase in MXEs given more sequencing data, we fit the sub-sampling data to the number of expected MXEs f(x) using Matlab. The green lines show the optimal fit for the expected number of validated MXEs in relation to the percentage of total RNA-seq reads used for validation (dark green 3 SJs 1 read; light green 3 SJs 3 reads). The actual measured data points are highlighted as yellow asterisks. The orange lines show the optimal fit for the expected number of initially 'validated MXEs' that will be rejected with increasing amounts of reads (dark orange 3 SJs 1 read; light orange 3 SJs 3 reads). The actual measured data points are highlighted as dark asterisks. Grey dashed lines indicate the predicted number of MXEs using 50, 100, 150, or 200% of the data (numbers are highlighted in the corresponding colors). Given a two-fold increase in the number of reads (100% – 200%), the expected number of validated MXEs (1SJ) is 1769 +/- 47 (95% confidence interval), validated MXEs (3SJ) is 1081 +/- 12, rejected MXEs (1SJ) is 227 +/- 9, and the number of rejected MXEs (3SJ) is 95 +/- 5. While the number of validated MXEs is far from saturation (a 100% increase in data results in 27% increase in the number of validations) the number of rejected MXEs seems to be saturated (a 100% increase in data results in 2% increase in the number of rejections).

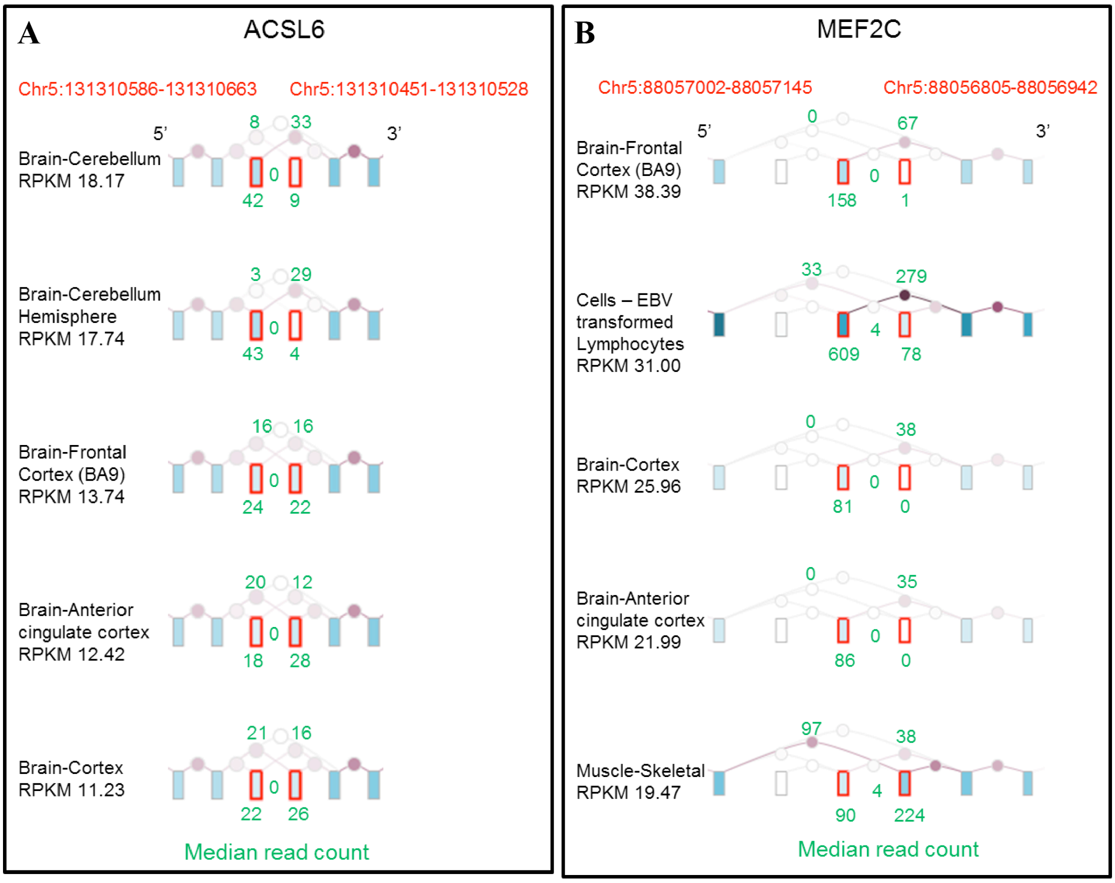

Appendix Fig. S12: GTEX validation of MXEs with two annotated exons. To exclude the possibility that some MXEs are a result of mapping artifacts that are specific to the aligner and the setting that were used, we compared MXE clusters that contained two ‘annotated other splicing’ exons to GTEx portal results (https://www.gtexportal.org/home/). Panels A and B highlight two representative examples of MXEs that are annotated as ‘other splicing’. A) GTEx splice junction reads for the *ACSL6* MXE across several human tissues. B) GTEx splice junction reads for the *MEF2C* MXE across several human tissues. Of note, we could validate all MXEs with annotated exons using data that was mapped with a different read aligner (bowtie 2 versus STAR) and very different parameters.

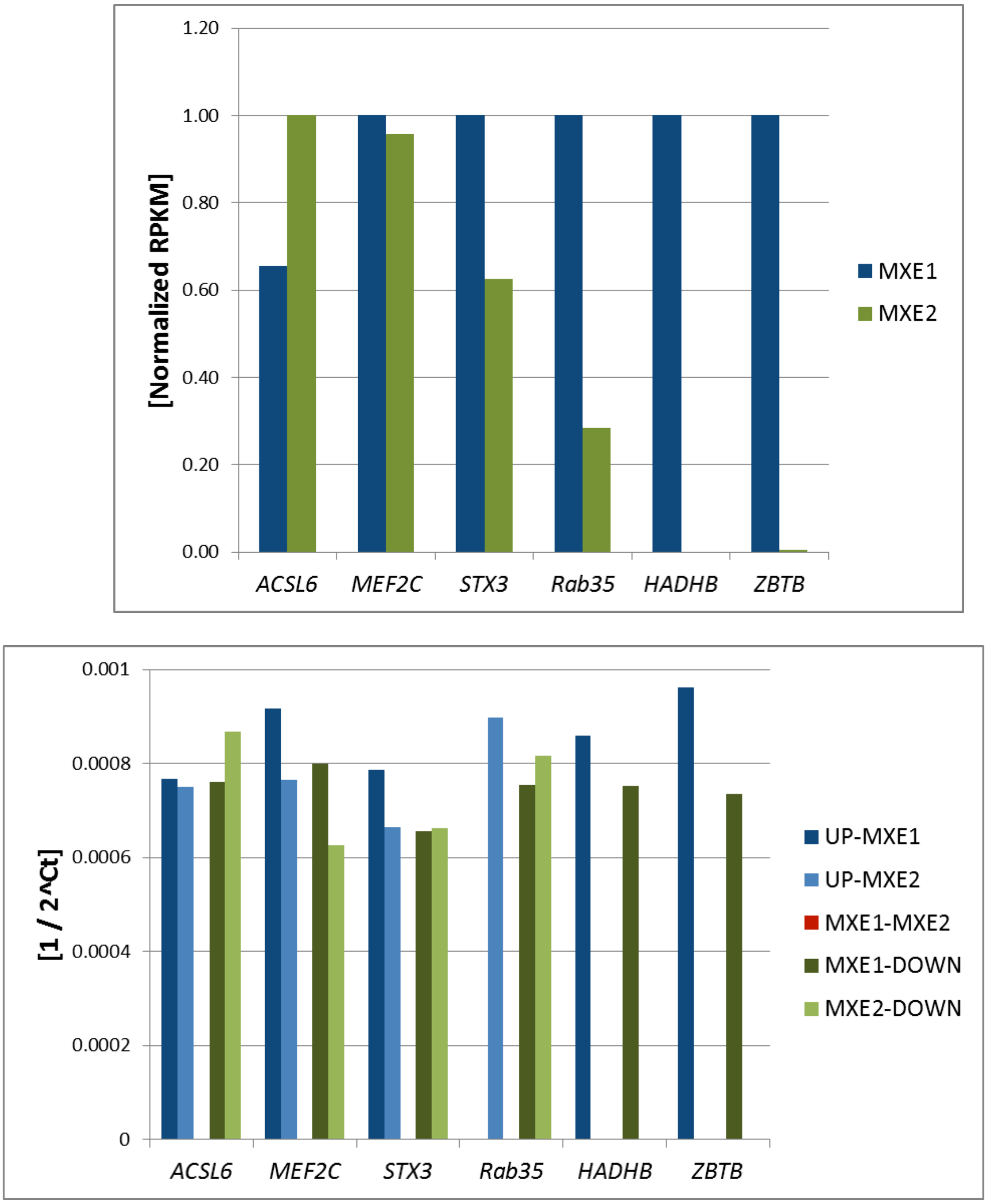

Appendix Fig. S13: qPCR validation of MXEs. To experimentally validate mutually exclusive splicing we selected 6 brain-expressed MXE clusters and validated their expression using qPCR on human brain-derived total RNA. **Upper panel:** The panel shows the normalized RPKM (maximum RPKM per gene equals 1) values for the MXEs of *ACSL6*, *MEF2C*, *STX3*, *Rab35*, *HADHB*, and *ZBTB*. Only *HADHB* and *ZBTB* show expression of only MXE1 while MXE2 is hardly spliced into brain transcripts. **Lower panel:** qPCR quantitation [1/2^Ct] using splice-junction bridging primers for 6 brain-expressed genes. While primer sets that bridge splice junctions to MXE up- (UP) or downstream (DOWN) exons show amplification, primer sets that bridge the two MXEs (MXE1-MXE2) show no amplification. The qPCR results almost perfectly reflect the RNA-seq-derived results, providing very strong evidence that the MXE candidates and novel exons are actually spliced mutually exclusively into transcripts. Of note, we were not able to design a functional qPCR primer for UP-MXE1 of *Rab35*.

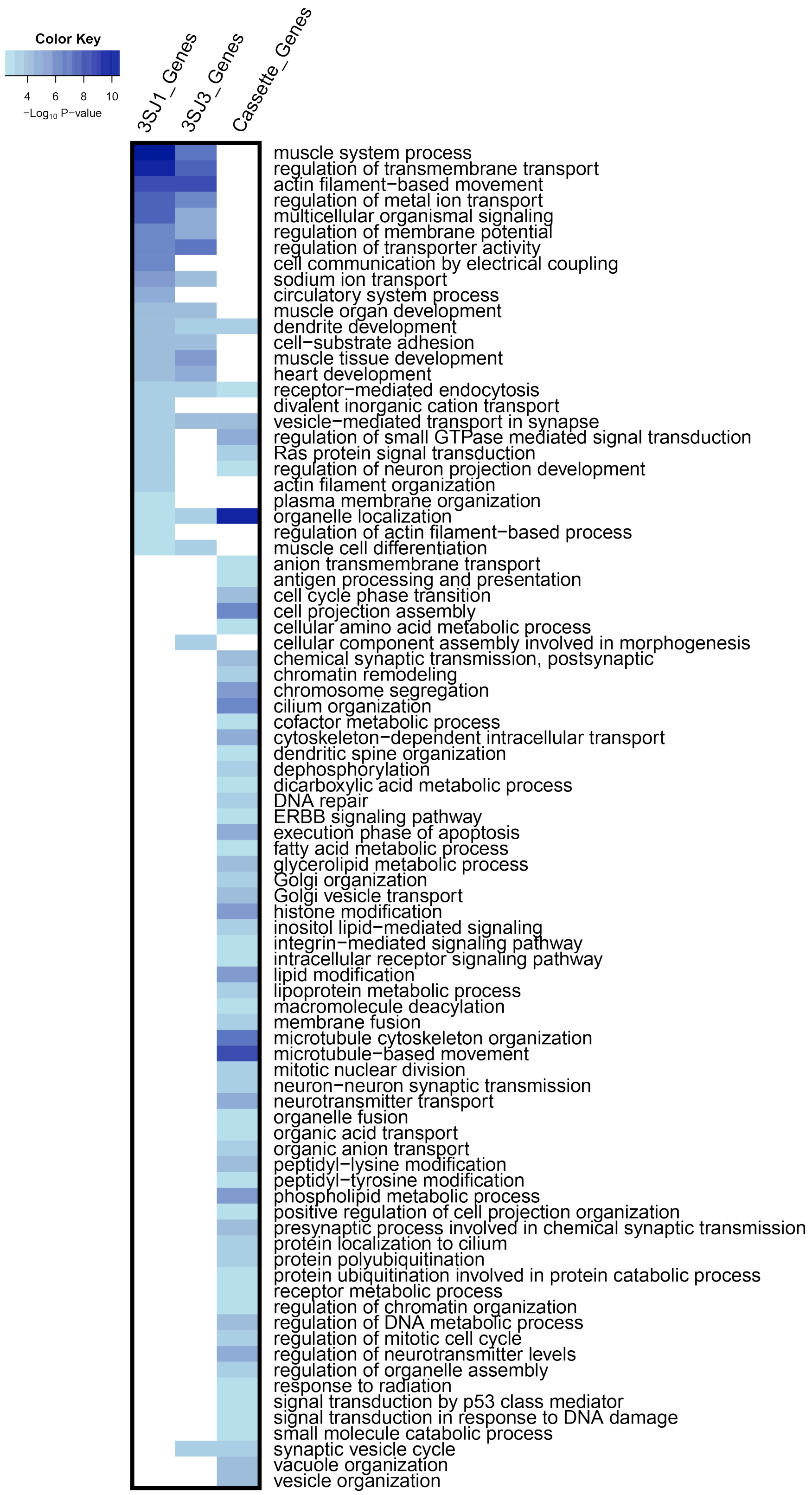

Appendix Fig. S14: MXE and cassette exon GO enrichment. Heatmap representation of significantly enriched GO terms for genes containing MXEs (3SJ1 and 3SJ3) or cassette exons. Whereas MXEs are strongly enriched for genes related to muscle and heart function and development, cassette exons show enrichment for organelle localization and microtubule-related terms.

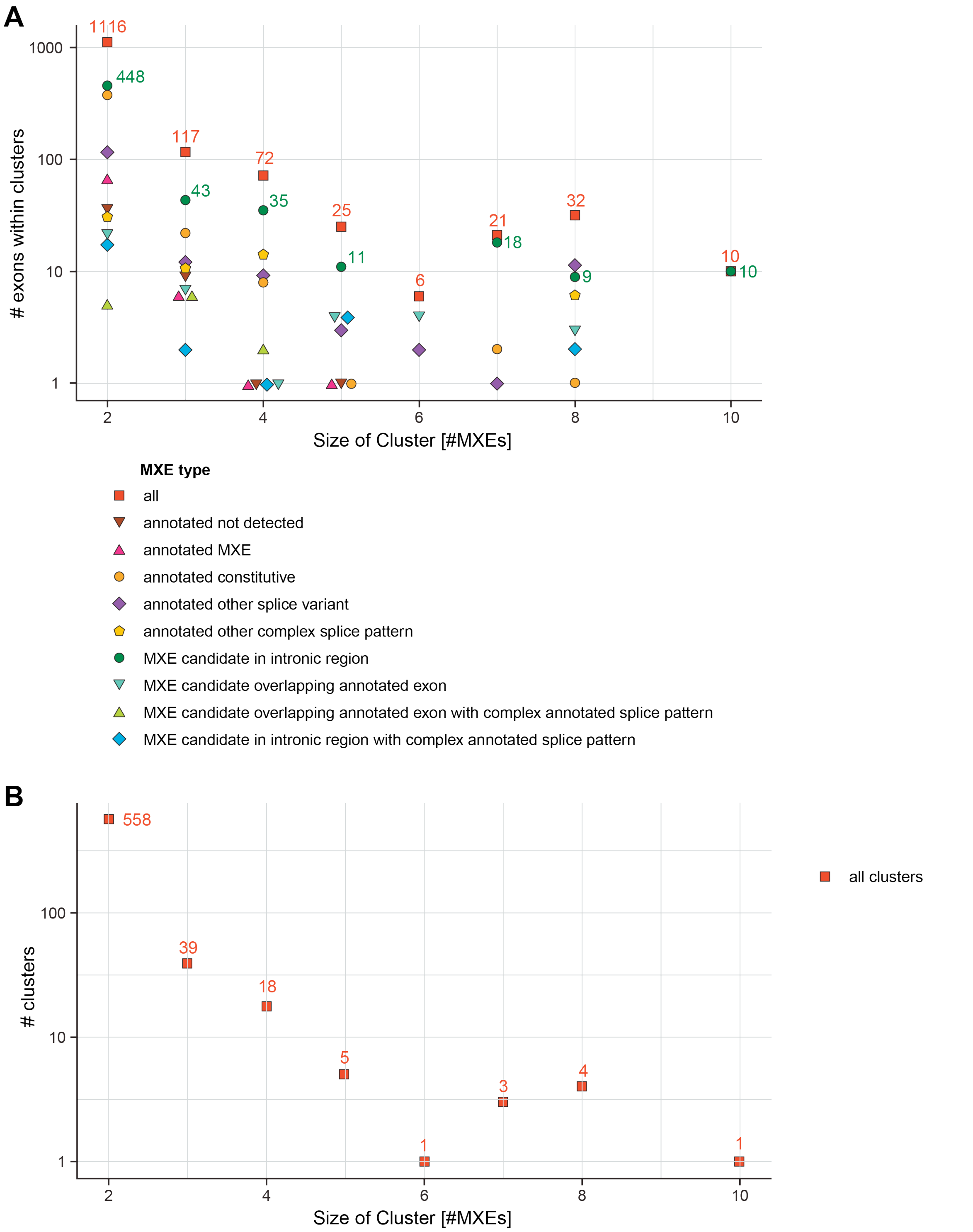

Appendix Fig. S15: Human genes with clusters of multiple MXEs. A) The scatter plots show the number of MXEs within clusters of certain size. The source of the MXEs within the clusters is given separately for the various annotated exon types (see Appendix Fig. S2). B) This scatter plot shows the number of clusters with a certain number of MXEs.

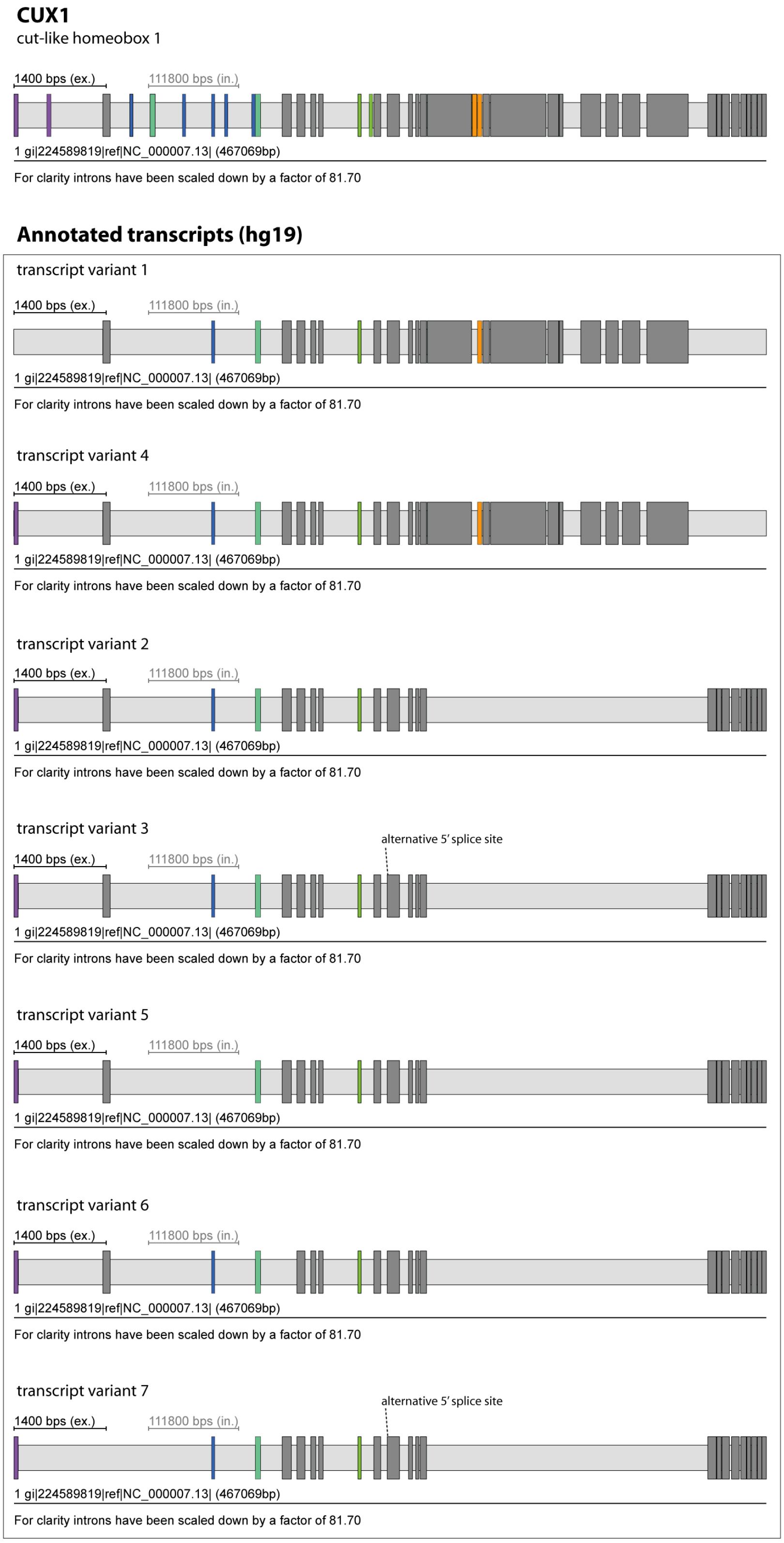

Appendix Fig. S16: Annotated *CUX1* isoforms with those exons highlighted that were validated as MXEs. All transcripts are represented 5' to 3'. Constitutive exons are shown as dark grey bars, and mutually exclusive exons are coloured with same colour for all MXEs of a cluster.

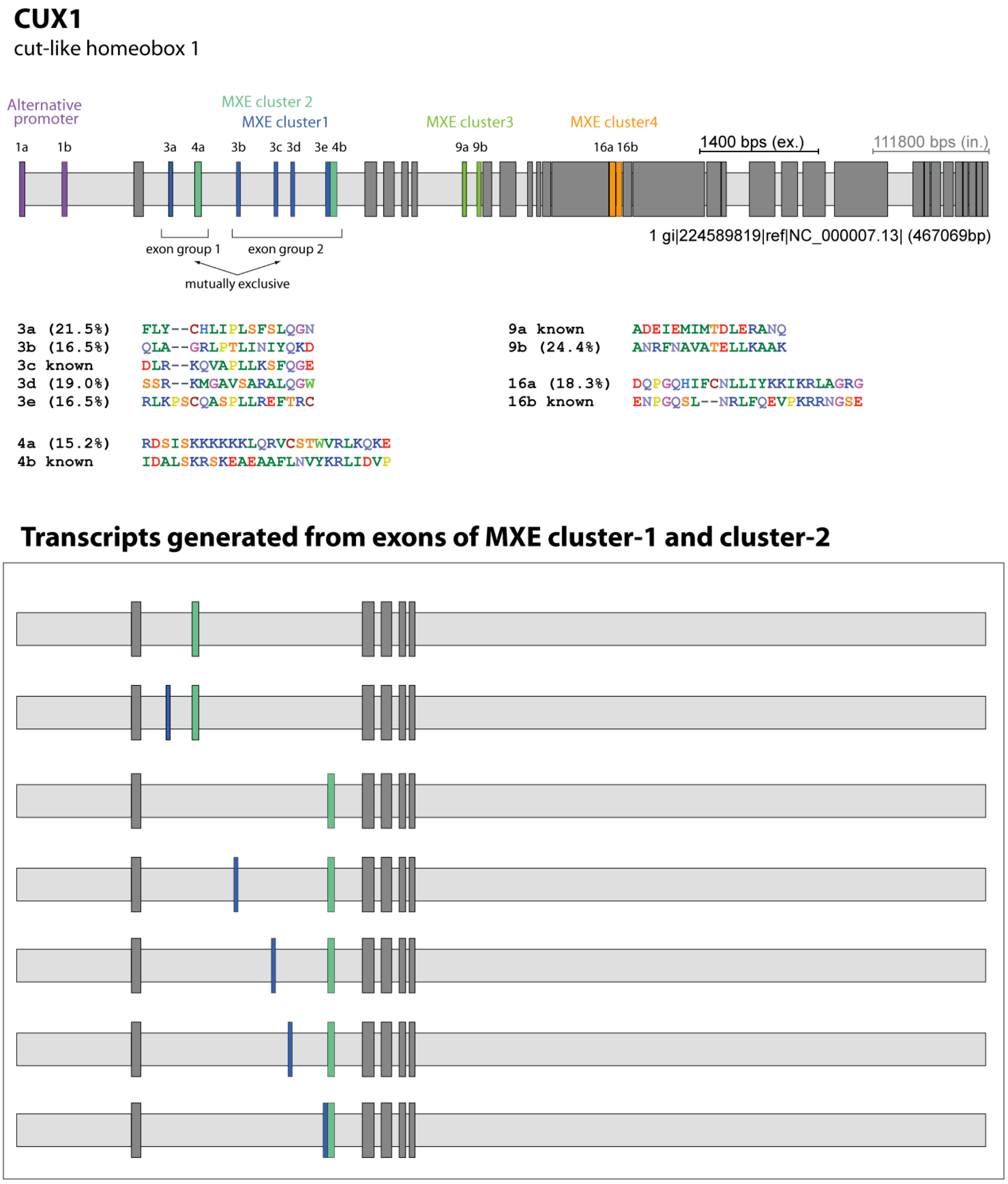

Appendix Fig. S17: MXE splicing of *CUX1*. Potential splice variants of the *CUX1* gene obtained by alternative splicing of MXE cluster 1 (dark blue exons) and MXE cluster 2 (dark green exons). MXE cluster 1 itself is differentially included. Amino acid sequences for the annotated and predicted exons are shown demonstrating their sequence similarity (coloured by chemical properties).

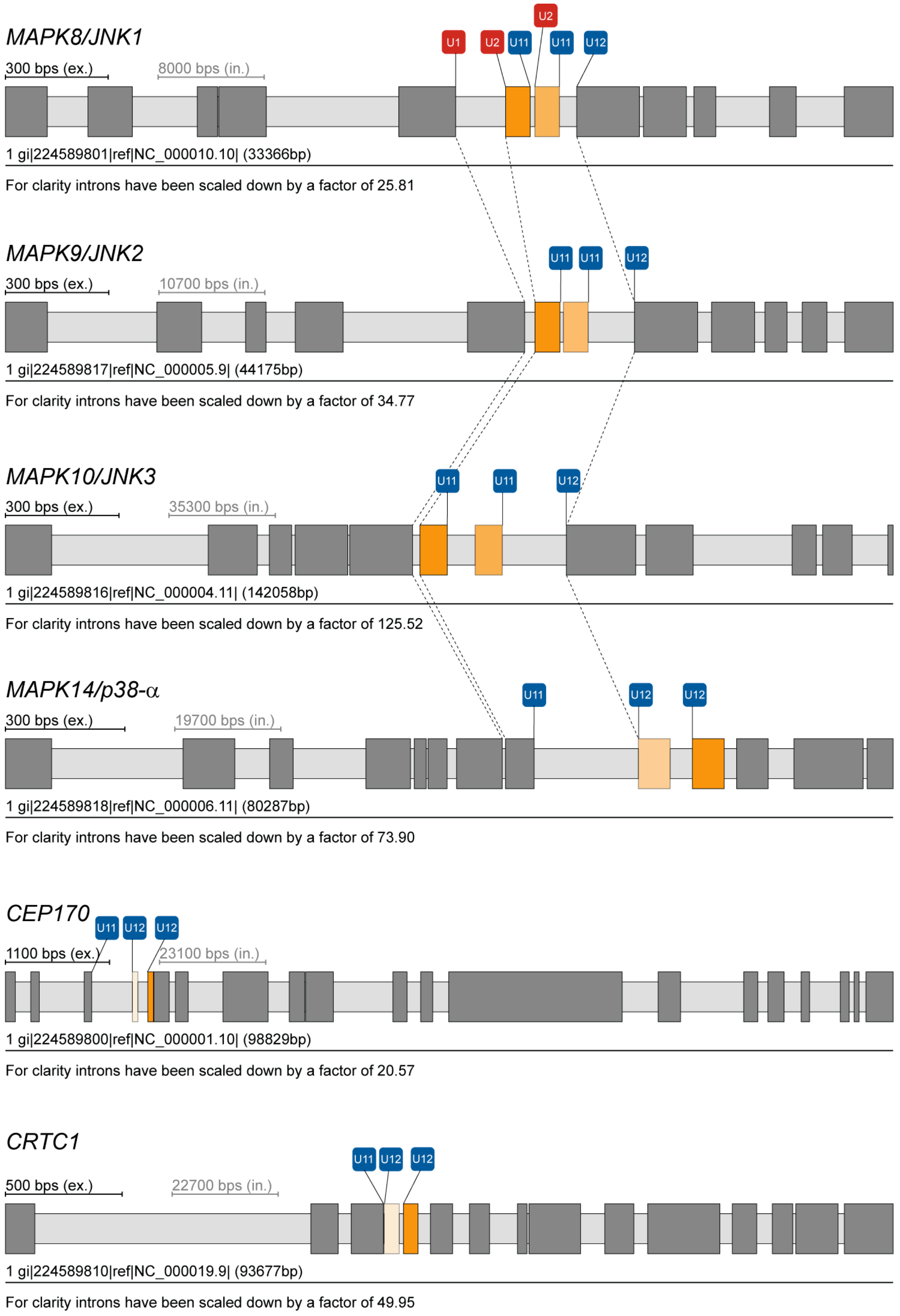

Appendix Fig. S18: U2/U12 incompatibility. In *MAPK8*, *MAPK9*, and *MAPK10* the U12 splice site is at the 3' end of the MXEs and in the other genes at the 5' site. While in *MAPK8*, *MAPK9*, and *MAPK10* the same exon has been duplicated (exon-6) indicating an origin of the cluster of MXEs before gene duplication happened, the corresponding following exon has been duplicated in *MAPK14* implying an independent exon duplication process. The *MAPK*

genes contain U12-type GT---AG introns, and *CEP170* and *CRTC1* contain AT---AC introns. The U12-type introns are found in all mammals but MXEs are not found for *MAPK10* in Metatheria, and not in other species for *CEP170* and *CRTC1*. *MAPK9* is not included in the list of MXE candidates because one of the candidate exons overlaps with a terminal exon in one of the *MAPK9* transcripts (see Appendix Fig. S2C). *MAPK9* is, therefore, included in the list of terminal MXE candidates (Appendix Fig. 4), of which many might not be terminal but internal exons. We suppose that the *MAPK9* transcript with the premature terminal exon is a mis-annotation, but computationally this case is treated as many other cases where the terminal exon might represent a bona-fide transcript termination.

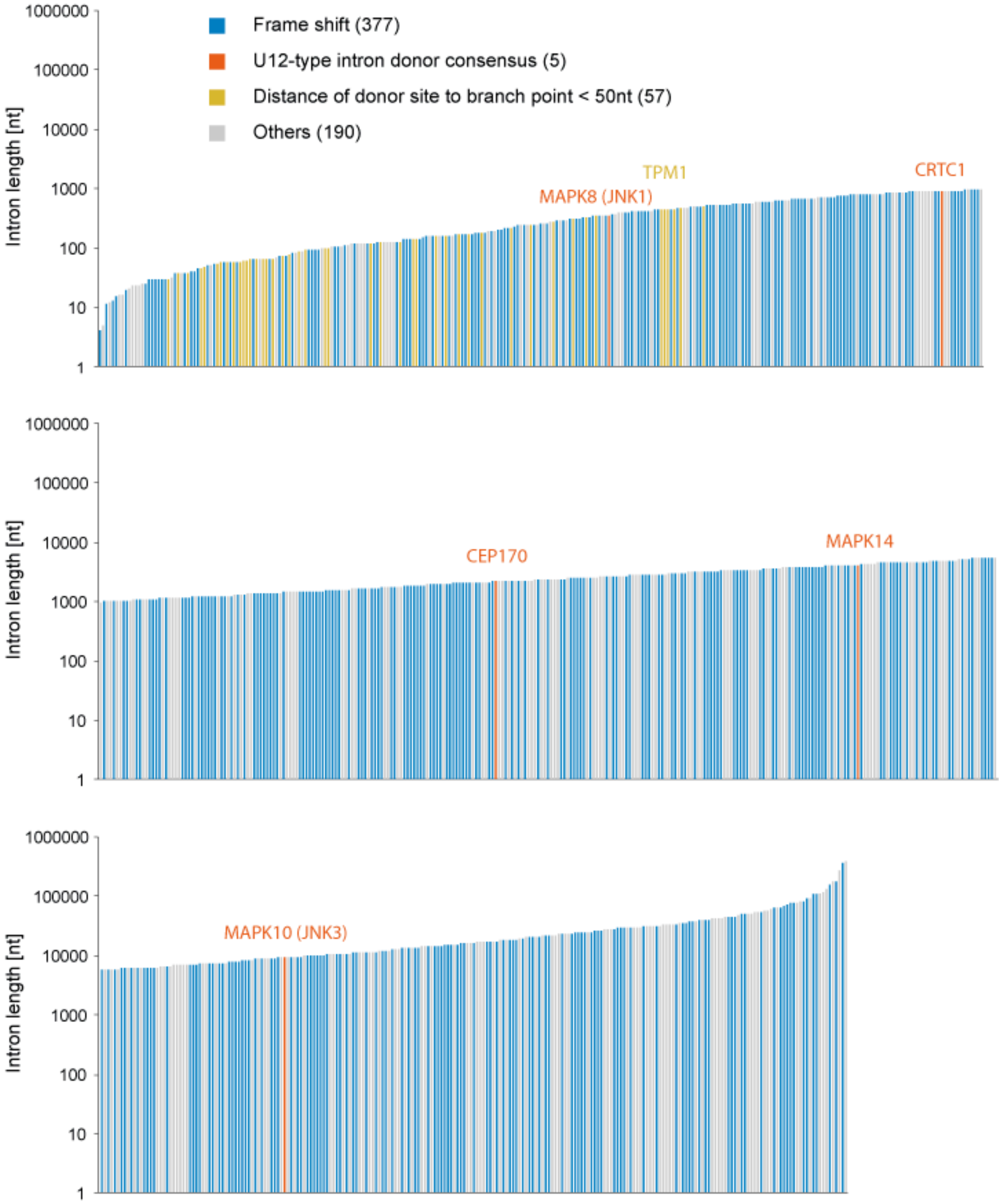

Appendix Fig. S19: Length distribution of the introns between MXEs. MXEs were sorted by increasing intron lengths. Introns are coloured if the mutually exclusive splicing of the respective MXEs is controlled by U2/U12-incompatibility (red), a close vicinity of the splice donor site and the branch point (yellow), and NMD (blue). Branch point predictions were limited to introns smaller than 500 bps. MAPK9 is not included in the list of MXE candidates because one of the candidate exons overlaps with a terminal exon in one of the *MAPK9* transcripts (see Appendix Figs. S2C and S18).

Reading frame (GFF/GTF file format definition)

```
5'   intron            exon              intron   3'
0     ...AG AGG TGA CAC CGC AAG CCT TAT ATT AGC GT...    0
1     ..AGA AGG TGA CAC CGC AAG CCT TAT ATT AGC AAGT.    1
2     .AGAA AGG TGA CAC CGC AAG CCT TAT ATT AGC AGT..    2
```

frame		5'		
		0	1	2
	0	114	89	49
3'	1	77	112	45
	2	66	51	28

Appendix Fig. S20: Analysis of MXE reading frame. Combined inclusion or exclusion of the exons of MXE clusters will not result in functional mRNAs if the exon lengths are not a multiple of three. This has been demonstrated for several cases, for example the *CACNA1C* gene coding for the L-type $Ca_V1.2$ calcium channel, where the inclusion or exclusion of both MXEs into the transcript did not result in functional protein (Tang et al. 2011). Curiously, MXE-joining reads were found for 91 (75%) of the annotated MXEs but only 25 (4%) of the predicted MXEs (Appendix Figs. S3A and S3D). More than ten exon-joining reads were found for 77% of these annotated MXEs indicating that NMD targeting is the major mode of excluding the translation of transcripts with multiple MXEs in these cases. While skipped exons are highly biased towards being symmetric thus preserving reading frame (Magen and Ast 2005) we only found a moderate reading frame bias for MXEs supporting the different roles of skipped and mutually exclusive exons in changing and modulating protein functionality, respectively.

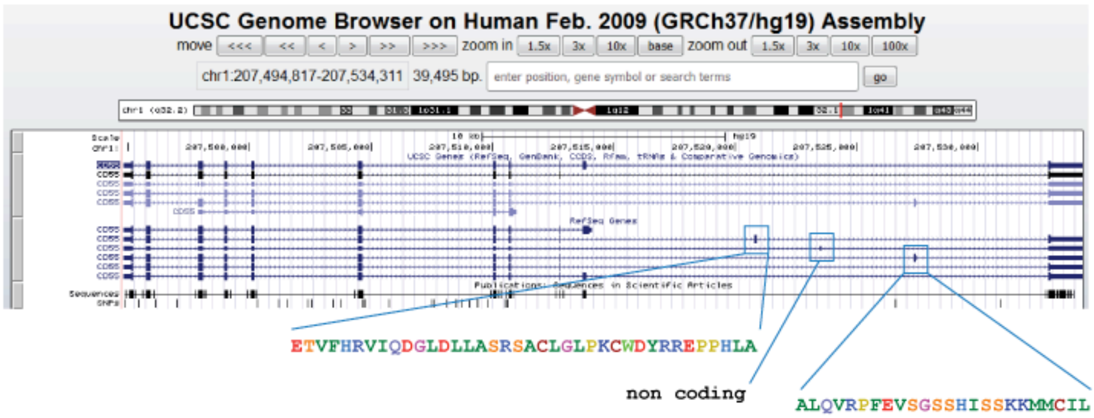

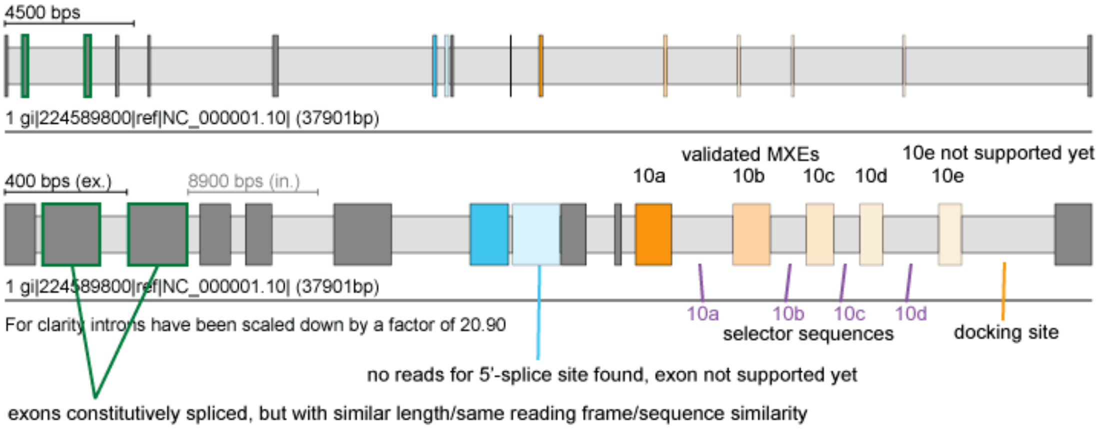


```
                                10        20        30        40
                          ....|....|....|....|....|....|....|....|.
10a:Reference             GSRPVTQAGMRWCDRSSLQSRTPGFKRSFHFSL-PSSWYYR
10b:41.07 %               ESHSVTQPGVQWRNLSSLQPLPPKFKRFSHLSLLSSSWDYR
10c:22.77 %               ---------VQWRDLGSLQALPPGFTPFSCLSL-PSSWDYR
10d:16.96 %               ESHSVTQAGVQWHDLGSLQPPSPGFK---------------
10e:16.07 %               GSHSVTQAGVQWCNLGSRQPLFPRLK---------------
```

```
                      10        20        30        40        50        60        70
              ....|....|....|....|....|....|....|....|....|....|....|....|....|....|...
              ...............((((.((((.........((((.((.((((..(((((((..--.(((...-(((..((((..
selector 10a  -TTTTTGAAATTTATTTTT-TGTAGAGACAGGATTTTCCTATGTTGCCCAGGCTG--GTTTCAA-ACTCCTGGCC-
selector 10b  -TTTTTG----TATTTTT-AATAGAGACAGGGTTTCACCATGTTGGCCAGTCTG--GTCTCGA-ACTCCTGACCT
selector 10c  TTTTTTG----TATTTTTCAGTAGAGACGGTGTTTCACCGTGTTATCCAGGATG--GTCTTGA-TCTCCTGGCCT
selector 10d  -TTTTTG----TATTTCT-AGTAGAGATGAGGTTTCACCATGTTGGCCAAGCTA--GTCTCAA-ACTCCTGACCT

docking site  ------ACAACAACAAAA-TTATAGACT-CACGAA-TGATACA--CGGTTCGTTACAAGATTCGTGA--ACTGTCCA
              -------........))))-))))......-..))))-)).))))--)))))))......)))....)))--))))....
```

Pan troglodytes, from cross-species search

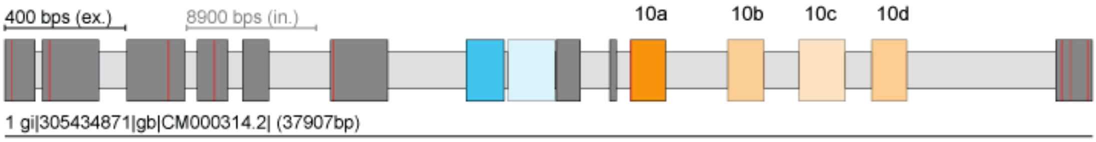

For clarity introns have been scaled down by a factor of 20.66

```
                   10        20        30        40        50
              ....|....|....|....|....|....|....|....|....|....|...
10a           ------------DSRPVTQAGMRWCDRSSLQSRTPGFKRSFHFSL-PSSWYYR
10b:46.43 %   ------------ESHSVTQPGVQWRNLSSLQPLPPKFKRFSHLSLPSSSWDYR
10c:26.34 %   GKMGKHFFFFQTESRSVAQAGVQWRHLGSLQALPPRFTPFSCLSL-PSSWDYR
10d:49.11 %   ------------ESHSVTQAGVQWHDLGSLQPPSPGFKRFSCLSL-PSSYDYR
```

Appendix Fig. S21: Example highlighting several aspects of generating the MXE candidate list, MXE validation, and splicing mechanism. The human *CD55* gene contains three regions with annotated and predicted exons matching the criteria of MXEs (sequence similarity, reading frame conservation, genomic vicinity). These exons are all in the MXE candidate list. RNA-seq read mapping showed that the two exons at the 5'-end of *CD55* (exons 2 and 3) are constitutively spliced exons, and that there are not enough reads yet to validate the splicing of exons 7b and 10e. These still have to be regarded as MXE candidates (=> missing data subtype). Although the human genome annotation available at UCSC shows some exons in the region of the exon10 MXE cluster, these exons do not match or even overlap with the novel exons predicted by our approach (upper panel). The predicted MXEs of the exon10 MXE cluster show considerable sequence homology, and this MXE cluster has also been identified in the chimpanzee (*Pan troglodytes*) genome assembly indicating that it likely evolved before separation of humans and chimpanzees. The splicing of the exon10 cluster might be regulated by competing RNA secondary structure elements. Here, the docking site was found in the intron between MXE candidate 10e and the following constitutive exon 11. The selector sequences were found downstream of each exon 10 variant.

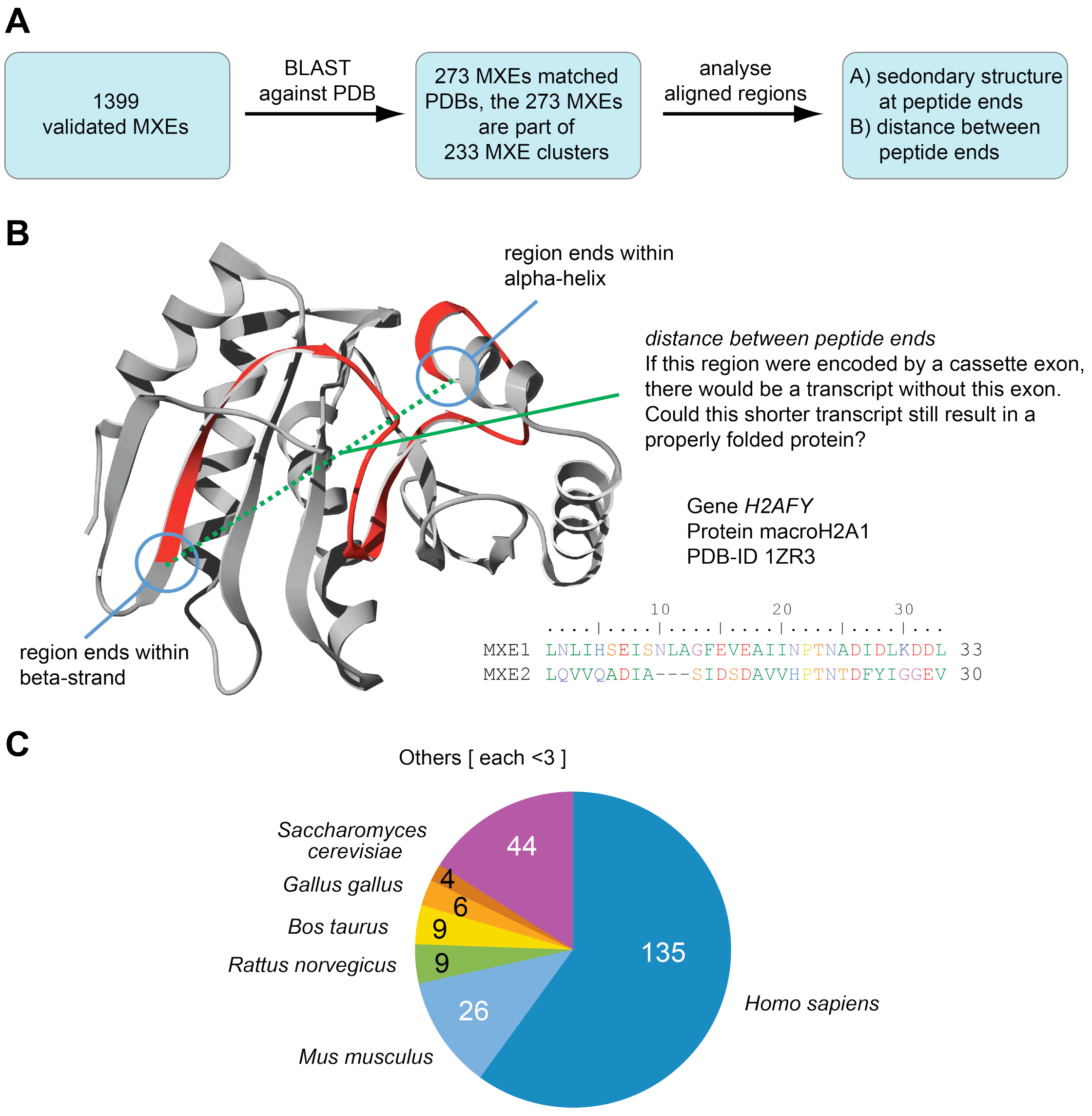

Appendix Fig. S22: Protein structure analysis of the MXE-encoded regions. A) To identify the best structural models for the sequences encoded by the MXEs we mapped the protein sequences of the respective genes against available protein structure data. Based on the database references in the PDB entries, the full-length proteins were downloaded from UniProt or GenBank and the corresponding gene structures of the eukaryotic proteins reconstructed with WebScipio. BLAST+ was used to search for the most similar UniProt/GenBank protein sequence compared to the human proteins containing MXEs. The hit with the lowest E-value was taken and the associated PDB chains were aligned to the human protein using m-coffee. The MXE part of the alignment was extracted for further analysis. As "intron distances" we determined the distances between the CA-atoms of the first and the last residues of the MXE-structures. B) Structural model of macroH2A1 (*H2AFY* gene) with the region encoded by the MXE coloured in red (PDB-ID 1ZR3). The parameters to distinguish potential encoding of protein regions by MXEs or cassette exons are shown on top of the structure. It is highly unlikely that a region ending in conserved secondary structural elements could be encoded by a cassette exon because the absence of this region would lead to a highly disturbed if not unfolded structure. Similarly, if the ends of the alternative exon encoded regions are far apart, it is unlikely that a structure missing this

region (in case the region would be encoded by a cassette exon) could fold correctly. C) Organismal distribution of PDB structures with mapped MXEs. The source organisms of the matched structures were compiled and plotted.

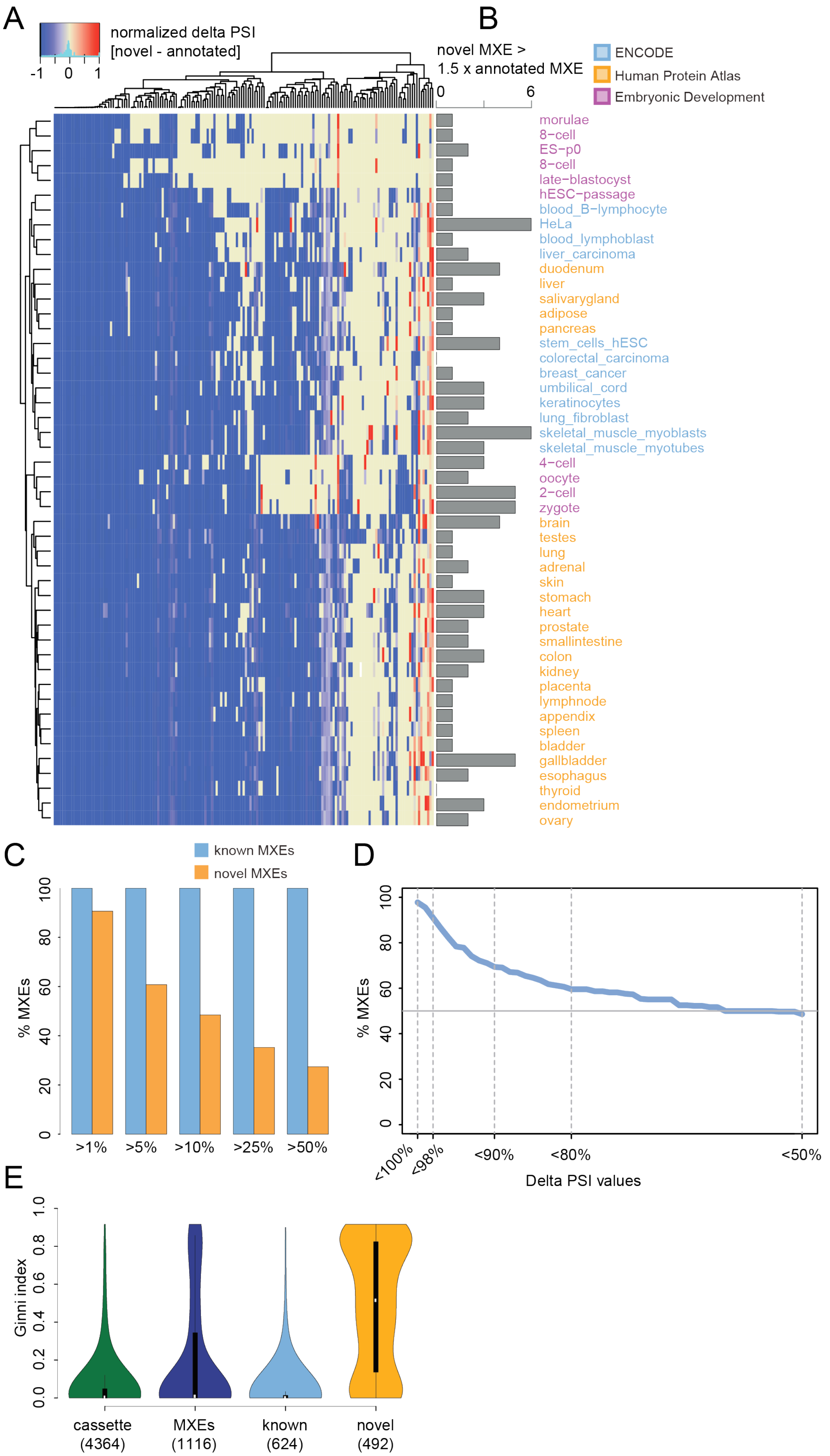
A
normalized delta PSI
[novel - annotated]
-1 0 1
B
novel MXE >
1.5 x annotated MXE
0 6
ENCODE
Human Protein Atlas
Embryonic Development
morulae
8−cell
ES−p0
8−cell
late−blastocyst
hESC−passage
blood_B−lymphocyte
HeLa
blood_lymphoblast
liver_carcinoma
duodenum
liver
salivarygland
adipose
pancreas
stem_cells_hESC
colorectal_carcinoma
breast_cancer
umbilical_cord
keratinocytes
lung_fibroblast
skeletal_muscle_myoblasts
skeletal_muscle_myotubes
4−cell
oocyte
2−cell
zygote
brain
testes
lung
adrenal
skin
stomach
heart
prostate
smallintestine
colon
kidney
placenta
lymphnode
appendix
spleen
bladder
gallbladder
esophagus
thyroid
endometrium
ovary
C
known MXEs
novel MXEs
% MXEs
0 20 40 60 80 100
>1% >5% >10% >25% >50%
D
% MXEs
0 20 40 60 80 100
<100% <98% <90% <80% <50%
Delta PSI values
E
Ginni index
0.0 0.2 0.4 0.6 0.8 1.0
cassette (4364)
MXEs (1116)
known (624)
novel (492)

Appendix Fig. S23: Annotated and novel MXE cluster expression. A) Heatmap showing the delta PSI (percent-spliced-in, see Material and Methods) values of all differentially expressed MXE clusters with at least 3 RPKM over different human tissues and developmental stages. Each pair of MXEs consists of one annotated and one novel exon. Delta PSI values were computed by subtracting the PSI value of the known MXE from the PSI value of the novel MXE. These values were scaled between -1 (high PSI for known MXE) and 1 (high PSI for novel MXE). B) The bar graph shows the sum of MXEs per tissue or state where the novel MXE is 1.5 fold higher expressed than the known MXE. C) Bar plot showing the percentage of known (blue) and novel MXEs (orange) with more than 1%, 5%, 10%, 25% and 50% of the MXEs having PSI values in at least one RNA-seq sample. We considered all the MXE pairs where one exon is known/annotated and the other is novel/intronic (214 MXE pairs). D) Delta PSI values for all 558 MXE pairs (1116 MXEs). For each MXE pair the delta PSI value was calculated and the percentage of MXE pairs was plotted (<100% to <50% in at least one sample). E) Gini index distribution for cassette exons (4364 exons), all MXE pairs in this study (1116), all annotated/known MXEs (624), and all novel/intronic MXEs (492). The Gini index for the cassette exons was calculated using the ENCODE dataset and cassette exon annotations were downloaded from the UCSC genome browser "knownalt" table.

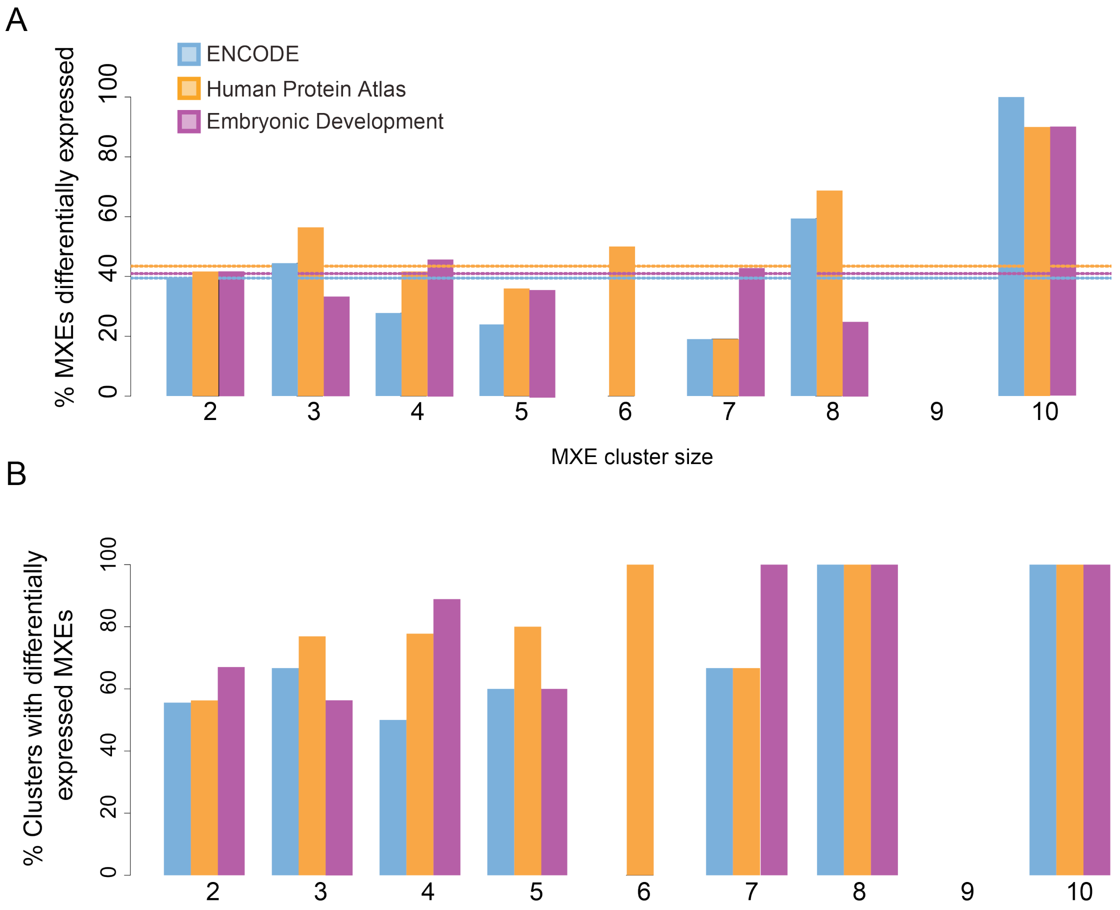

Appendix Fig. S24: Percentage of differentially expressed MXEs per cluster. A) Bargraph showing the percentage of differentially expressed MXEs per dataset sorted by MXE cluster size. The percentage refers to the total number of validated MXEs in the particular cluster. The dashed lines represent the sum of observed differentially expressed MXEs per sequencing project. B) Similar to A), but this time showing the percentage of MXE clusters, for which differentially expressed MXEs have been observed.

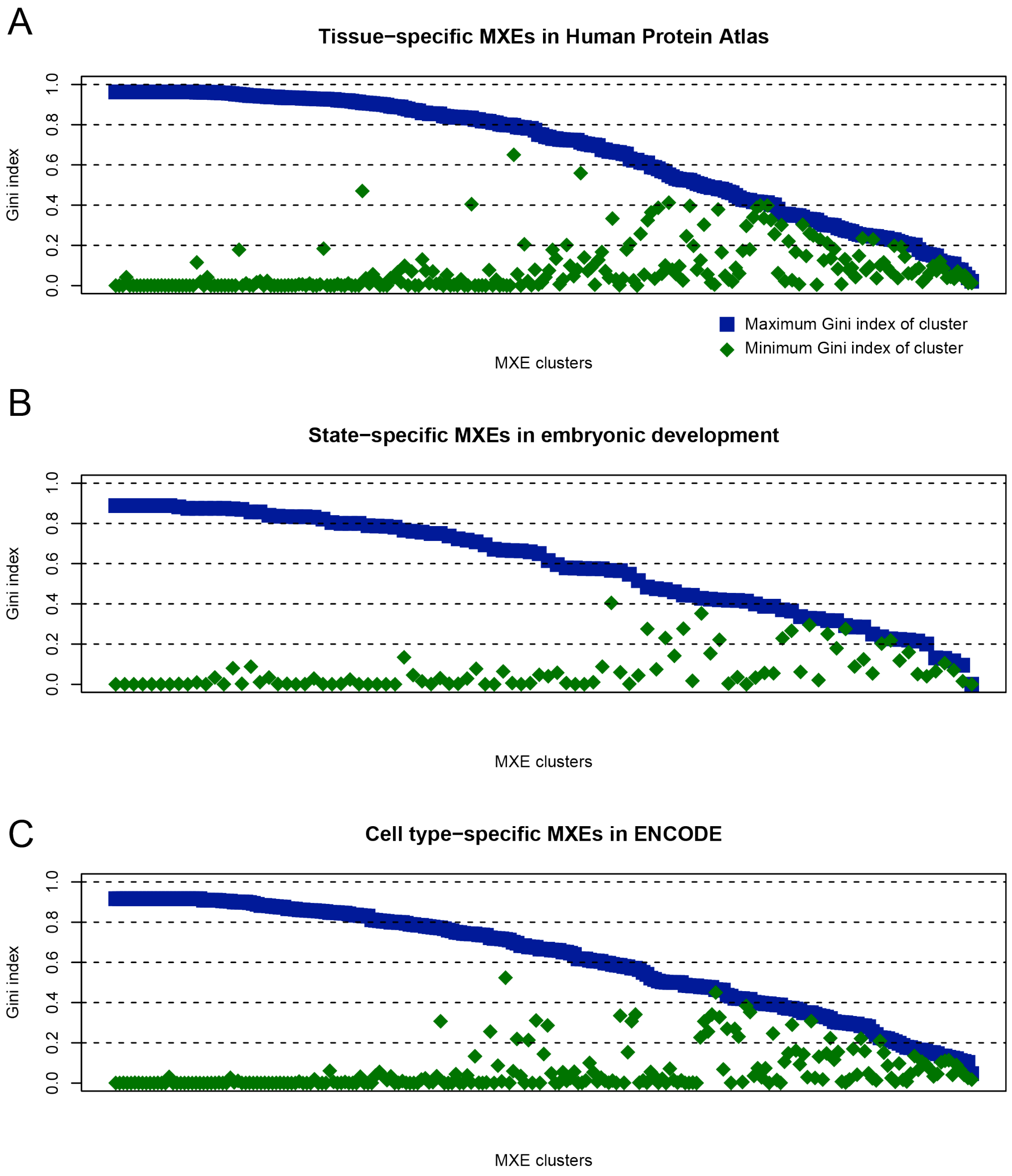

Appendix Fig. S25: MXE expression-specificity. Plots showing expression-specificity for MXEs clusters for the Human Protein Atlas (A), embryonic development (B), and ENCODE (C) datasets as measured by the Gini index. The Gini index is a measure for inequality in the data, a high Gini Index signifies high inequality or high specificity (few outstanding high values and the rest is low, Gini >0.5) and a low Gini Index low inequality or low specificity (all values are within the same range, Gini <0.5). The Gini index was calculated based on the mean PSI value per group for each MXE. Only MXE clusters in which at least two MXEs have a mean RPKM ≥ 10 in at least one group were selected. Finally, the two MXEs per cluster with the highest Gini value (blue squares) and the lowest Gini value (green diamonds) were selected for presentation.

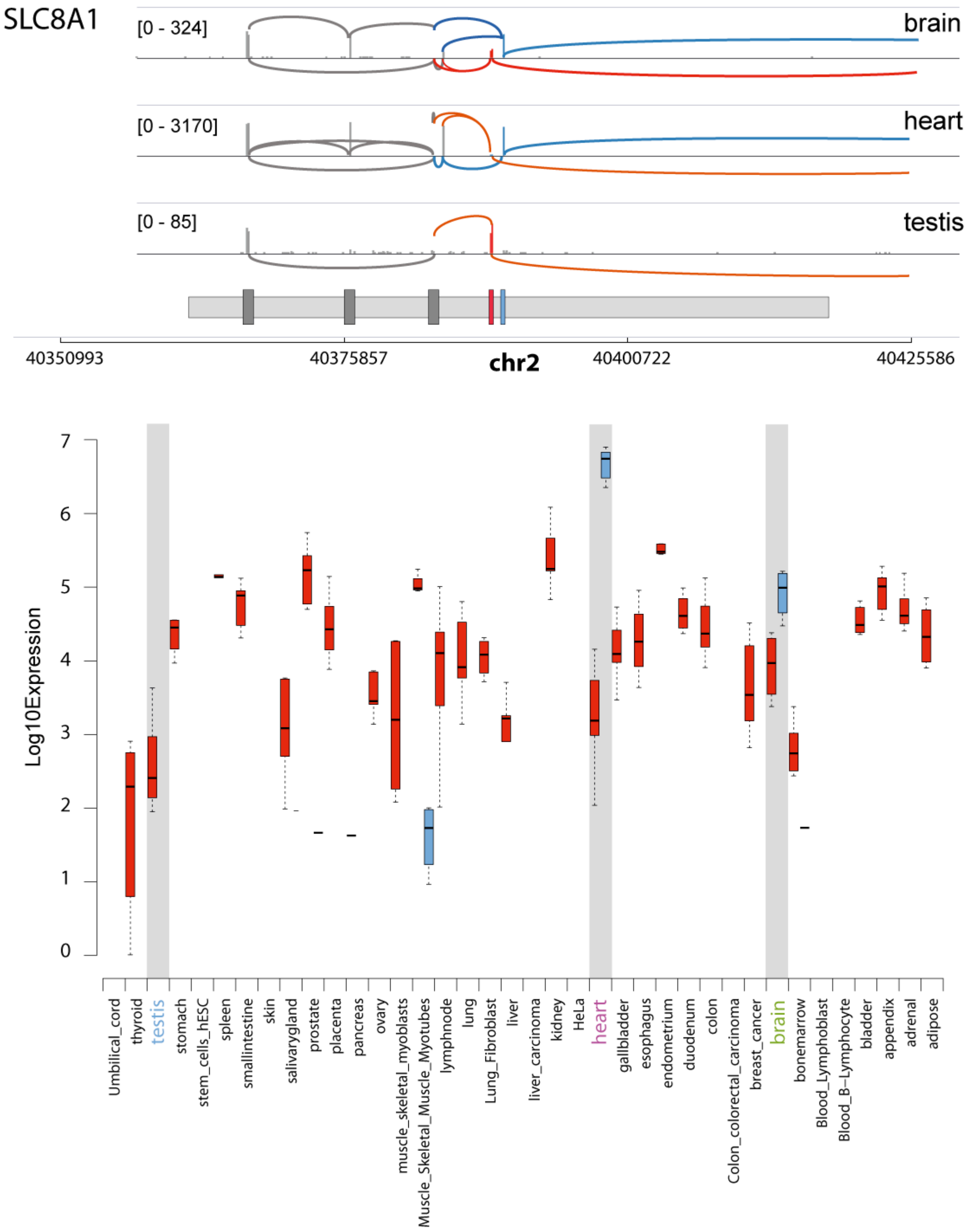

Appendix Fig. S26A: The Solute Carrier Family 8 Member A1 gene (*SLC8A1*) is an example of an MXE cluster with a large differences between the Gini-values of the MXEs. Notably, exon-4b is not expressed, or expressed at extremely low levels, in most of tissues. Top) Sashimi plot showing the expression and splice-junction reads on the 5' exons of *SLC8A1*. Bottom) Box and whisker plots showing expression of exon-4a with low Gini-value (red; meaning ubiquitous expression) and exon-4b with high Gini-value (blue, meaning highly selective expression) across human tissues.

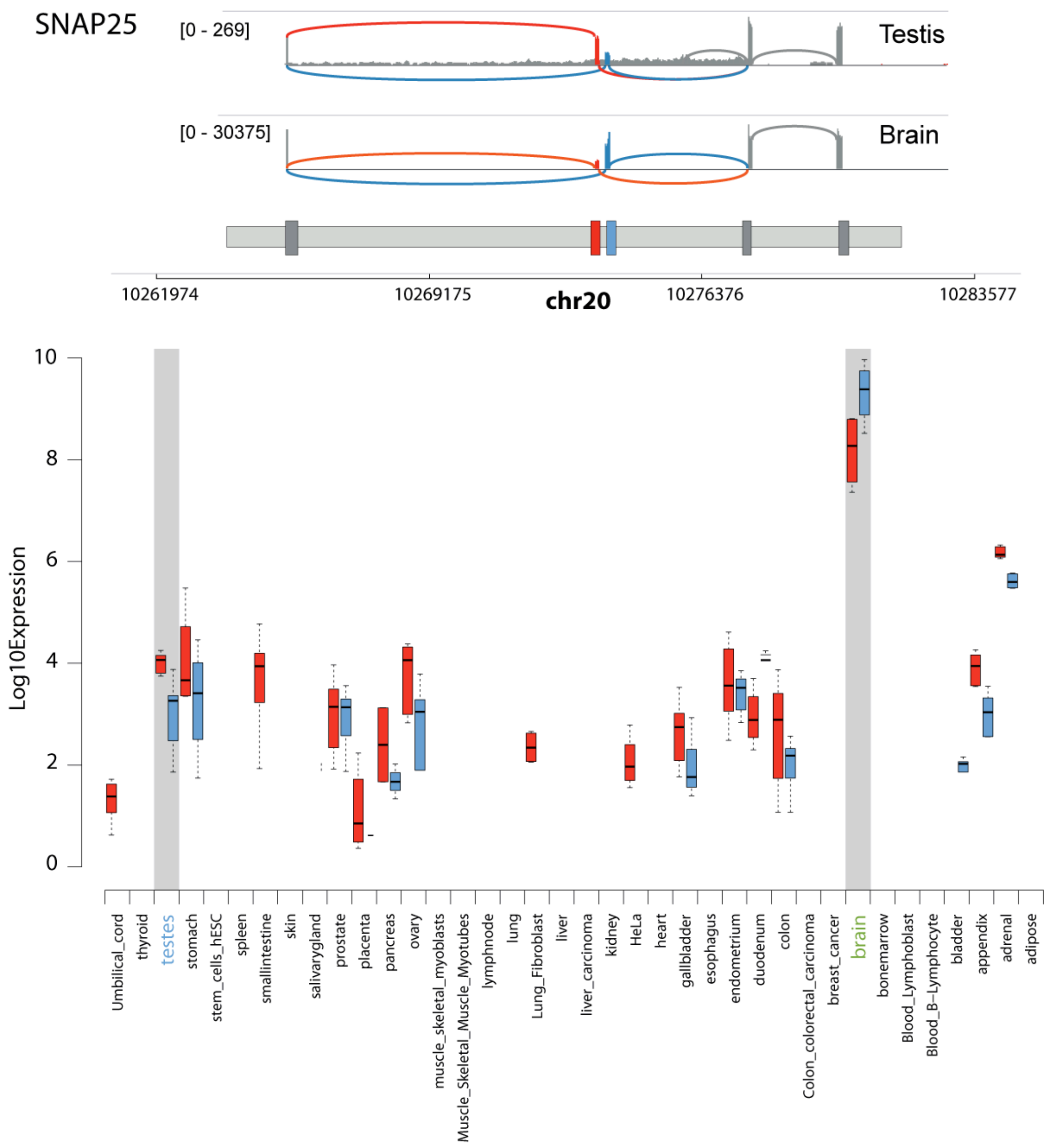

Appendix Fig. S26B: The Synaptosome Associated Protein 25 (*SNAP25*) is an example of an MXE cluster with a small differences between the Gini-values of both MXEs. Top) Sashimi plot showing the expression and splice-junction reads on all exons of *SNAP25*. Bottom) Box and whisker plots showing expression of exon-2a (red) and exon-2b (blue) across human tissues. In general, exons 2a and 2b are expressed in equal amounts in most tissues, and are highly enriched in brain.

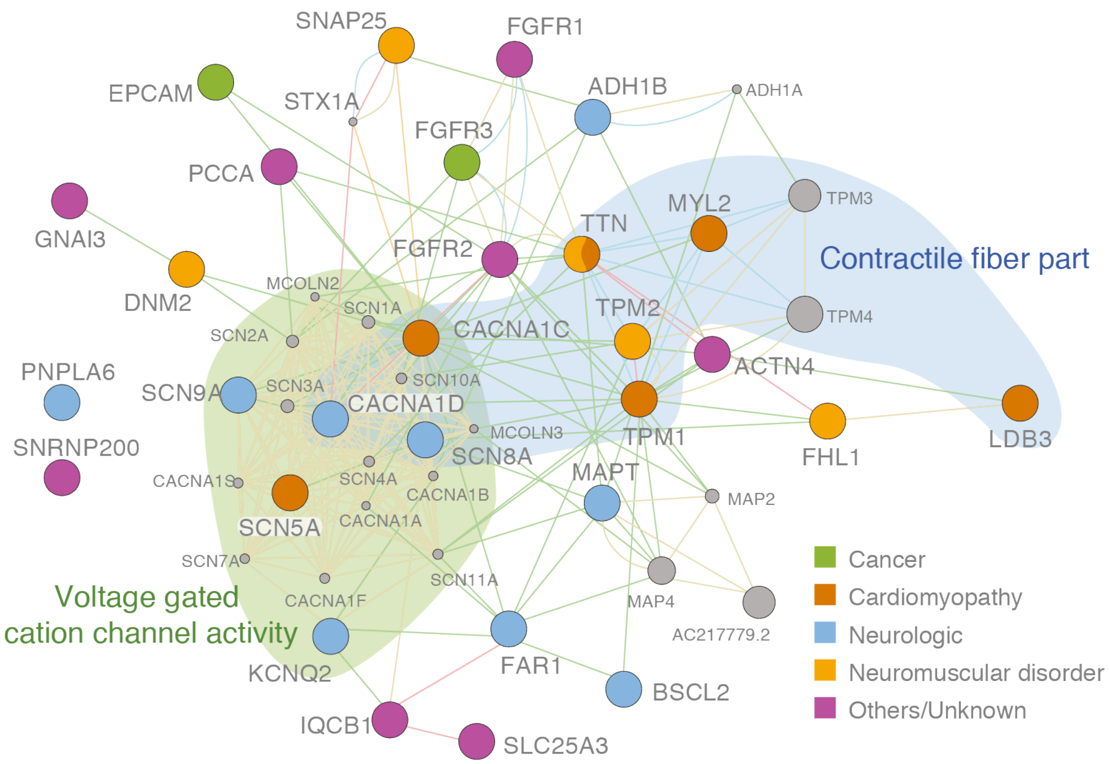

Appendix Fig. S27: Network plot of diseases, genes, and protein classes. The genes containing pathogenic SNPs in MXEs form a highly connected PPI network based on pathway, genetic, physical and protein domain interactions. Only two genes were not connected (*PNPLA6* and *SNRNP200*). Two GO terms were significant in the resulting network: 'contractile fiber part' (blue transparent shade) and 'voltage gated cation channel activity' (green transparent shade). The proteins are coloured based on the disease caused by the SNP (see legend).

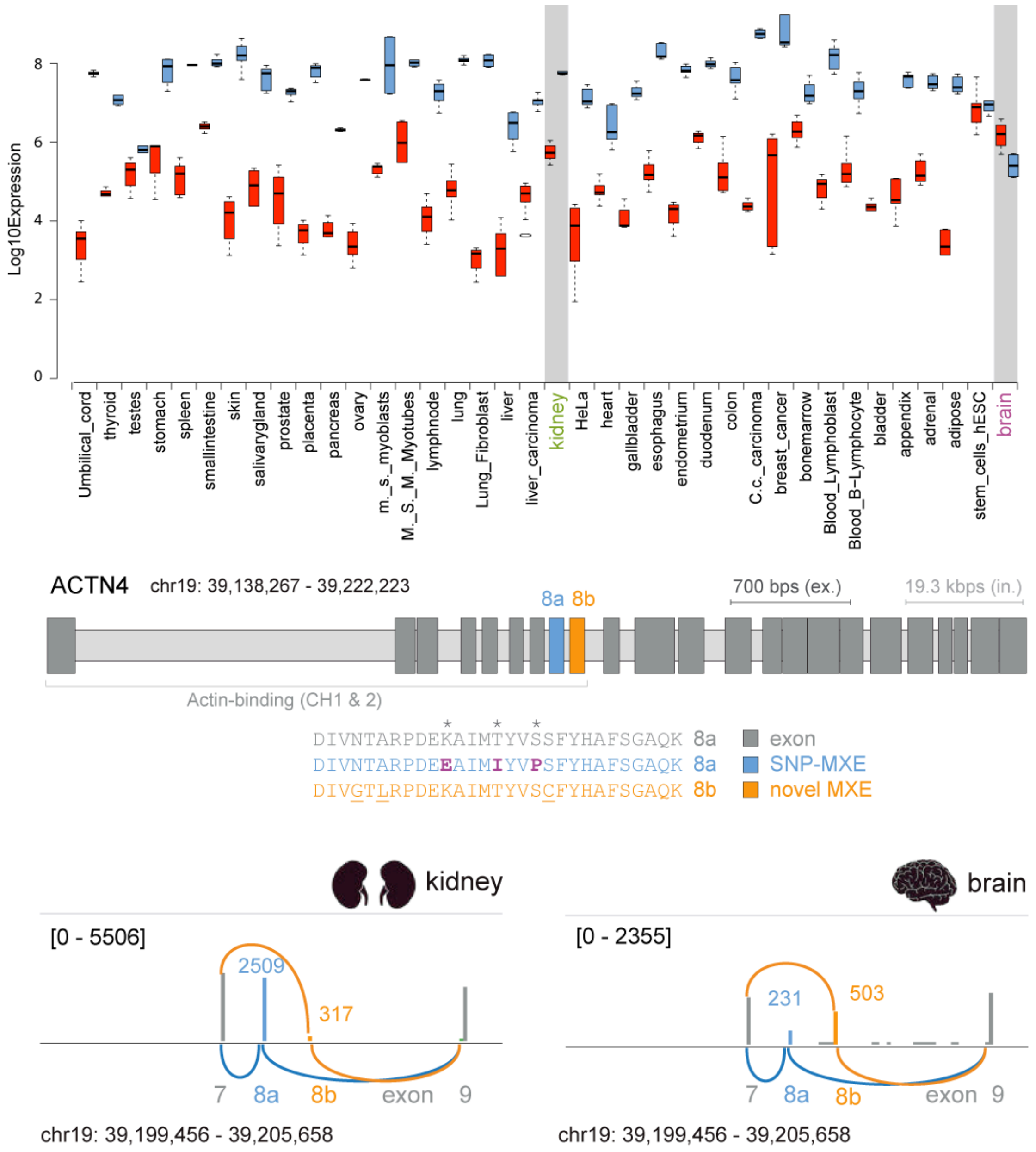

Appendix Fig. S28: Differential expression of and pathogenic mutations in MXEs of *ACTN4*. The α-actinin 4 *ACTN4* gene is an example of a gene carrying multiple pathogenic SNPs (Kaplan et al. 2000) in an exon, exon 8a, that we found to be part of a cluster of two mutually exclusive spliced exons (Lek et al. 2010). The two exons 8a and 8b of the *ACTN4* MXE cluster have distinct expression in kidney and brain. The 8a exon carrying the missense mutations is higher expressed in kidney, while exon 8b is higher expressed in brain. Strikingly, the reported missense mutations in exon 8a are causative for the kidney disease FSGS (familial focal segmental glomerulosclerosis).

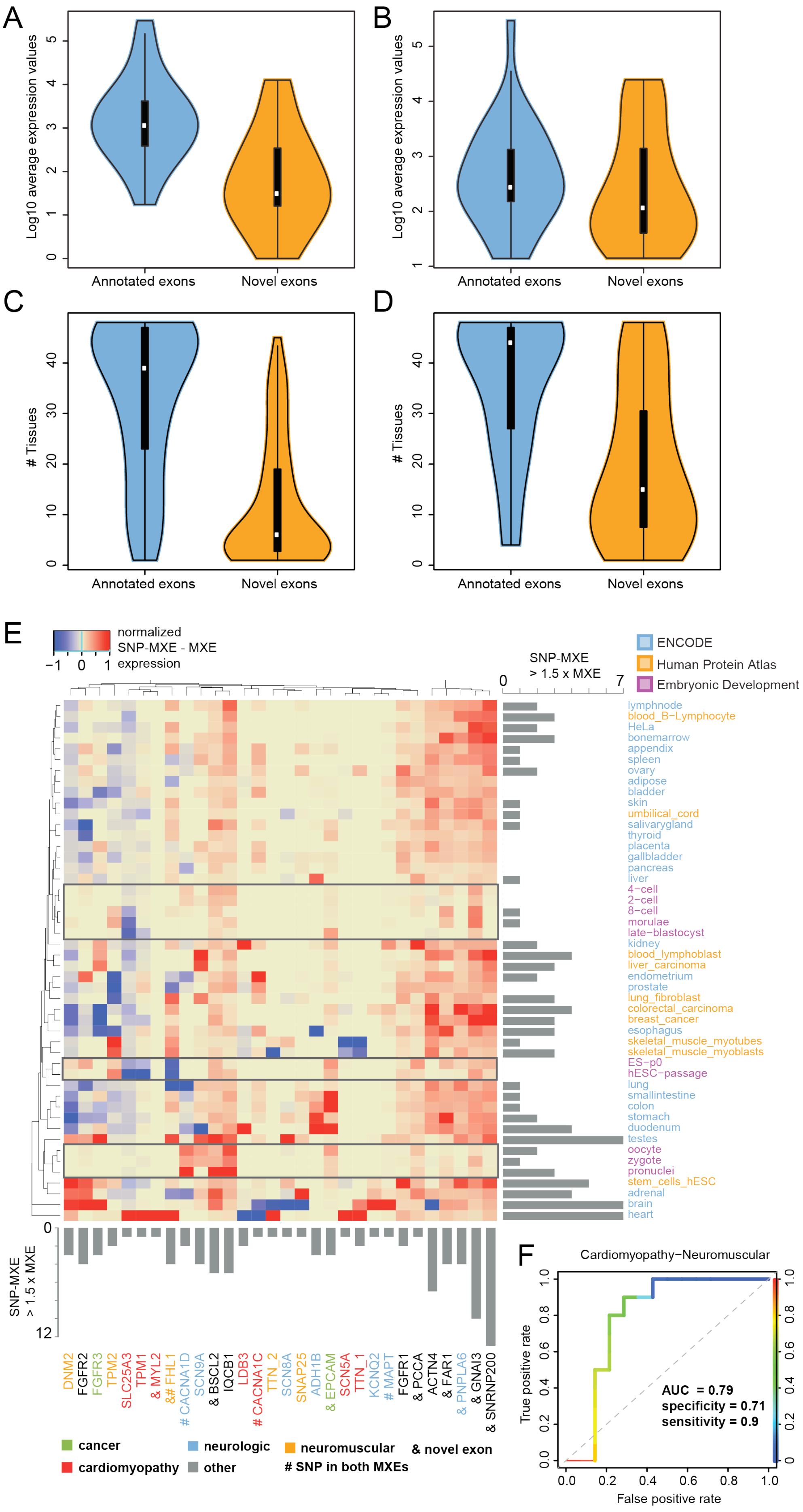
A
B
C
D
E
F
Log10 average expression values
Tissues
Annotated exons
Novel exons
normalized SNP-MXE - MXE expression
SNP-MXE > 1.5 x MXE
ENCODE
Human Protein Atlas
Embryonic Development
lymphnode
blood_B-Lymphocyte
HeLa
bonemarrow
appendix
spleen
ovary
adipose
bladder
skin
umbilical_cord
salivarygland
thyroid
placenta
gallbladder
pancreas
liver
4-cell
2-cell
8-cell
morulae
late-blastocyst
kidney
blood_lymphoblast
liver_carcinoma
endometrium
prostate
lung_fibroblast
colorectal_carcinoma
breast_cancer
esophagus
skeletal_muscle_myotubes
skeletal_muscle_myoblasts
ES-p0
hESC-passage
lung
smallintestine
colon
stomach
duodenum
testes
oocyte
zygote
pronuclei
stem_cells_hESC
adrenal
brain
heart
DNM2
FGFR2
FGFR3
TPM2
SLC25A3
TPM1
& MYL2
&# FHL1
CACNA1D
SCN9A
& BSCL2
IQCB1
LDB3
CACNA1C
TTN_2
SCN8A
SNAP25
ADH1B
& EPCAM
SCN5A
TTN_1
KCNQ2
MAPT
FGFR1
& PCCA
ACTN4
& FAR1
& PNPLA6
& GNAI3
& SNRNP200
cancer
neurologic
neuromuscular
& novel exon
cardiomyopathy
other
SNP in both MXEs
Cardiomyopathy-Neuromuscular
True positive rate
False positive rate
AUC = 0.79
specificity = 0.71
sensitivity = 0.9

Appendix Fig. S29: Comparison of the expression level and tissue distribution of known exons and their corresponding novel partner MXEs. All calculations in this figure are based on RPKM expression values and not PSI values. A) and B): Average expression values for each tissue were calculated for each MXE within an MXE cluster with at least one exon containing a pathogenic SNP and one exon without SNP. In chart A the maximum of the average expression per tissue for each SNP-MXE was calculated and compared to the expression of the partner MXE in the same tissue. The other way round (chart B), the maximum of the average expression per tissue for the novel MXEs was calculated and compared to the expression of the partner SNP-MXE in the same tissue. Accordingly, the novel exons are strongly expressed in selected sets of tissues, although their SNP-containing partner MXEs are still, on average, higher expressed. C) and D): Comparison of the tissue distribution of the annotated and novel exons. The number of tissues with observed expression was counted for each annotated exon (left plots) and each novel exon (right plots). Only tissues in which the MXEs have high average expression (RPKM > 3) were considered. In general, the annotated exons are expressed in multiple tissues while, in contrast, the novel exons are expressed in a small number of tissues. The left chart represents all MXEs (C), and the right chart the sub-selection of those MXEs with reported SNPs and their partner MXEs (D). E) Heatmap showing the expression difference of MXE clusters containing pathogenic SNPs scaled between -1 and 1 (blue = high expression non-SNP containing MXE, red = high expression SNP-containing MXE). Columns represent MXE clusters and rows tissues, cell types, and developmental stages. The column bar graph summarizes counts where the SNP-containing MXE is 1.5 fold more expressed than the non-SNP containing MXE, whereas the row bar graph shows this for each tissue, cell type, and developmental stage. F) Receiver operating characteristic (ROC) curve showing true and false positive rates for cardiomyopathy-neuromuscular disease prediction based on spatio-temporal MXE expression. To obtain at least ten observations per category with an expression > 3 RPKM, diseases were grouped into cardio-neuromuscular (n=10) and other diseases (n=14) and predicted using leave-one-out cross-validation with a Random Forest. Cardiac-neuromuscular diseases could be predicted with an accuracy of 79% (p-value < 0.03), a specificity of 71%, a sensitivity of 90% and an area under the ROC curve (AUC) of 79%. The predictive value of RPKM values is therefore very similar to that of delta PSI (Figs. 4C and 4D) and PSI values (data not shown).

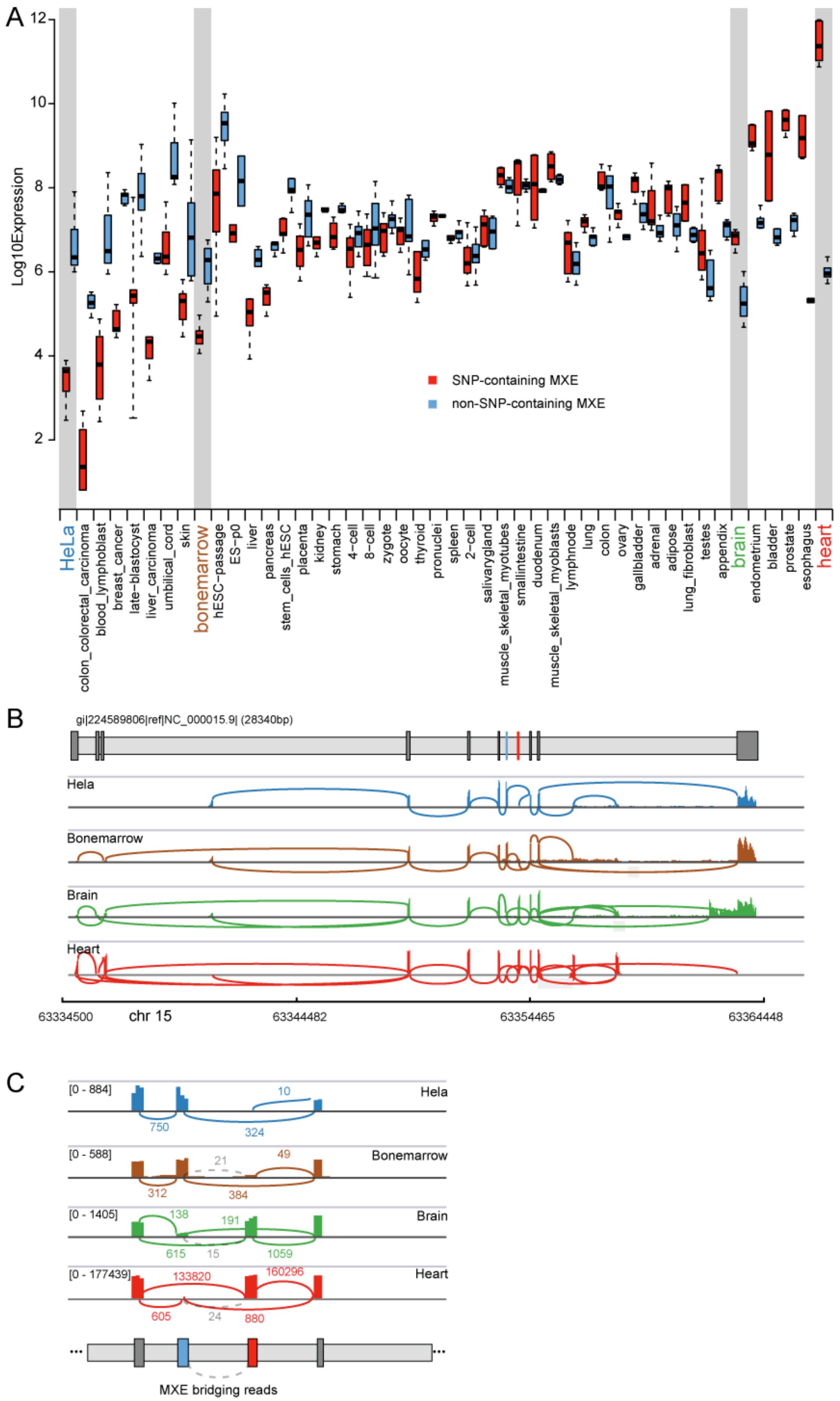
A
Log10Expression
SNP-containing MXE
non-SNP-containing MXE
HeLa
colon_colorectal_carcinoma
blood_lymphoblast
breast_cancer
late-blastocyst
liver_carcinoma
umbilical_cord
skin
bonemarrow
hESC-passage
ES-p0
liver
pancreas
stem_cells_hESC
placenta
kidney
stomach
4-cell
8-cell
zygote
oocyte
thyroid
pronuclei
spleen
2-cell
salivarygland
muscle_skeletal_myotubes
smallintestine
duodenum
muscle_skeletal_myoblasts
lymphnode
lung
colon
ovary
gallbladder
adrenal
adipose
lung_fibroblast
testes
appendix
brain
endometrium
bladder
prostate
esophagus
heart
B
gi|224589806|ref|NC_000015.9| (28340bp)
Hela
Bonemarrow
Brain
Heart
63334500
chr 15
63344482
63354465
63364448
C
[0 - 884]
Hela
[0 - 588]
Bonemarrow
[0 - 1405]
Brain
[0 - 177439]
Heart
MXE bridging reads

Appendix Fig. S30: *TPM1* MXE expression and splicing. The *TPM1* gene contains a pathogenic SNP in the exon 6a (red color in boxplots and exon in gene model) causing cardiomyopathy (Primary familial hypertrophic cardiomyopathy). This exon is part of an MXE cluster with another exon (exon 6b-blue boxplots and exon in gene model) not containing any annotated pathogenic SNP. A) Box and whisker plots showing expression of MXE without (blue) and with (red) pathogenic mutations across human tissues and development. In general, exons 6a and 6b are expressed in equal amounts in most tissues, whereas disease-relevant tissues show very high expression of the SNP-containing exon and very low expression of the non-pathogenic exon (e.g. heart). It is very important to note that high expression of the pathogenic exon in additional tissues suggests that the disease might also affect other organs besides heart (e.g. brain). B) Sashimi plot showing the expression and splice-junction reads on all exons of *TPM1*. Reads that span the two exons of the MXE cluster are shown in grey dashed lines, resulting in translational frameshift and non-functional protein. MXE with pathogenic SNP shown in red, without in blue. C) Zoom-in on the MXE cluster.

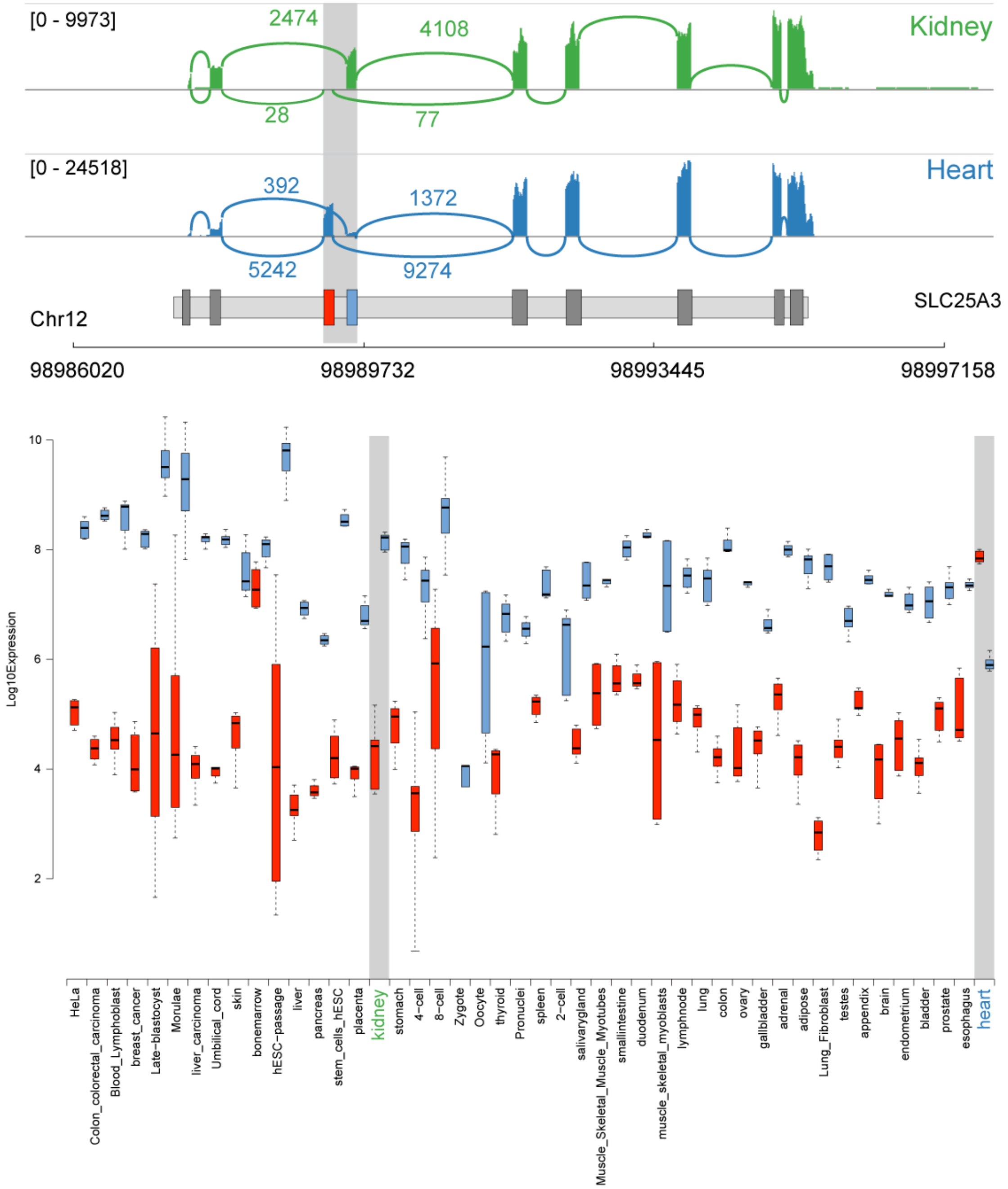

Appendix Fig. S31: *SLC25A3* MXE Expression. The *SLC25A3* gene contains a cluster of two MXEs, with exon 3a (red boxes) not containing any annotated pathogenic SNP, and exon 3b (blue boxes) with a pathogenic SNP causing a mitochondrial disease (Mitochondrial phosphate carrier deficiency). The expression of exon 3b is higher than that of exon 3a in all tissues, cell lines and embryonic development stages, except in heart, where the exon **not** carrying the pathogenic SNP turns out to be the predominant one. Interestingly, although our disease annotation categorizes the *SLC25A3* mutation as 'mitochondrial' the disease manifests itself as cardiomyopathy. This has implications for our classification, as *SLC25A3* is misclassified as 'cardiomyopathy' while being annotated as 'other' disease.

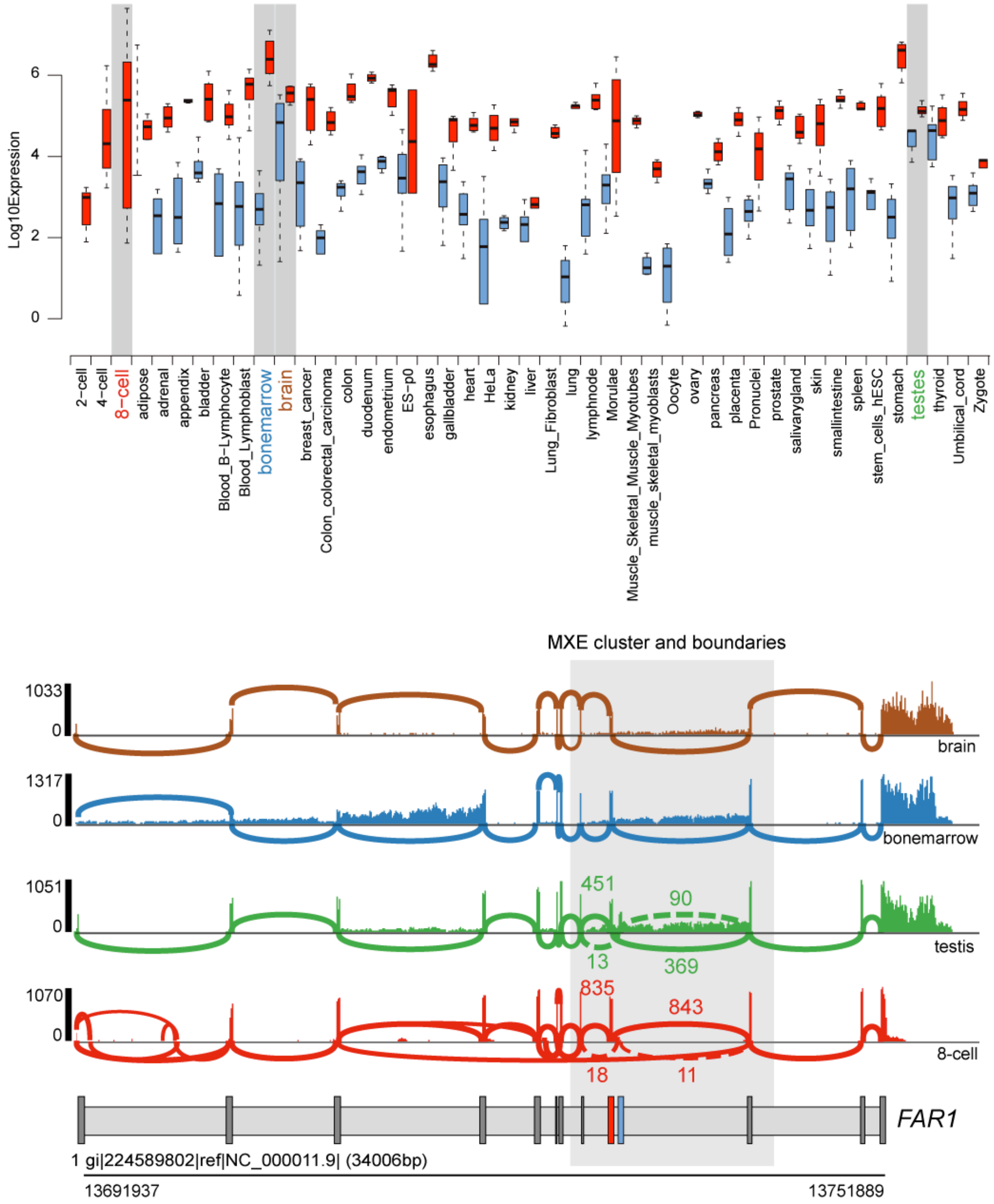

Appendix Fig. S32: *FAR1* MXE expression and splicing. The *FAR1* gene contains a pathogenic SNP in the annotated exon 9a (red) causing Rhizomelic chondrodysplasia punctata. This exon is part of an MXE cluster with a novel exon (exon 9b – blue) not containing any annotated pathogenic SNPs. In general, exons 9a and 9b are expressed in most tissues but exon 9a is on average expressed 8 times higher. Splice junction (SJ) reads for exon 9a are shown with solid lines and SJ reads for exon 9b with dashed lines.

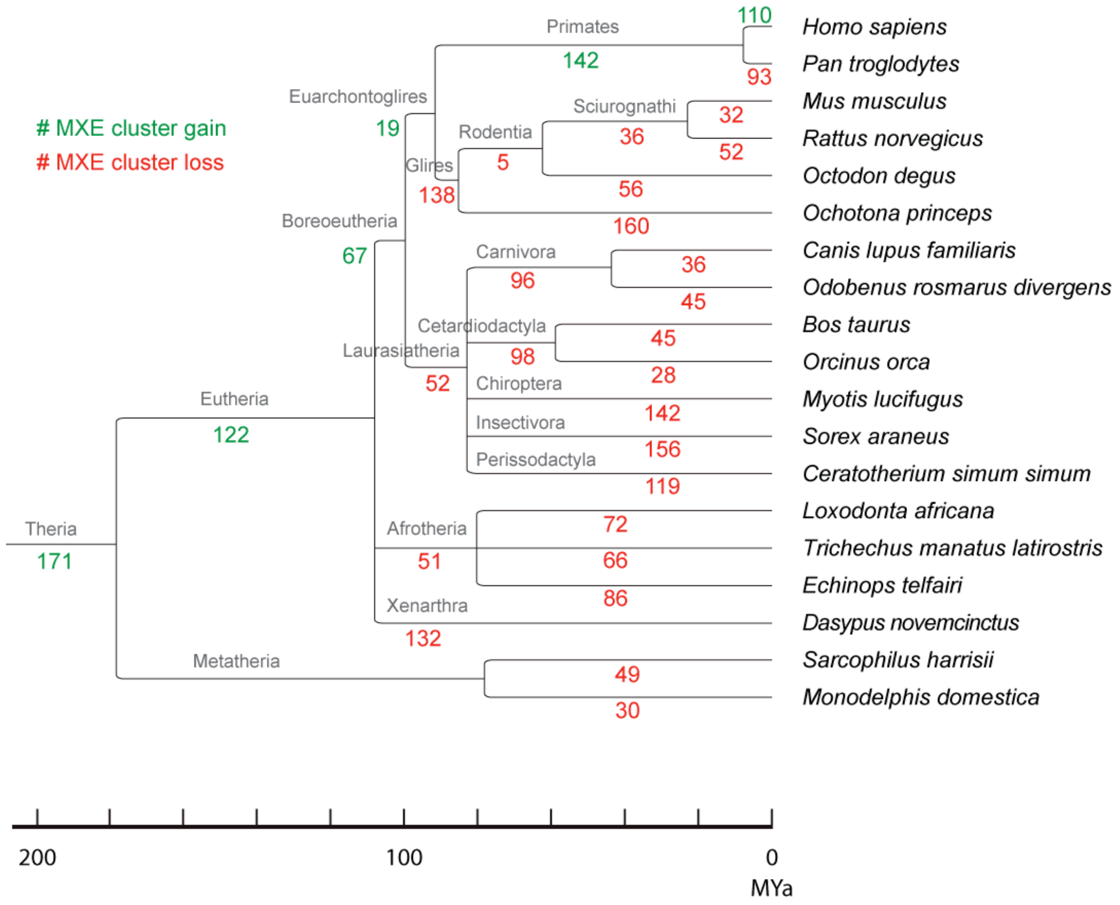

Appendix Fig. S33: Gain and loss of clusters of MXEs. The gain and loss of clusters of MXEs were plotted onto the evolutionary tree of the analysed 18 mammals. Human clusters of MXEs can be shared with any mammal and any combination of mammals resulting in theoretically 262,143 combinations. Of these, we identified only 267 demonstrating that most clusters are conserved in larger groups (e.g. in all Theria or Euteria), that in most cases clusters are lost in major branches, and that only a small part is lost species-specific. The *Ochotona princeps* genome assembly is far more fragmented than the other genome assemblies, which might explain the high number of species-specific MXE cluster loss events. Divergence time estimates were obtained from TimeTree (Hedges et al. 2006).

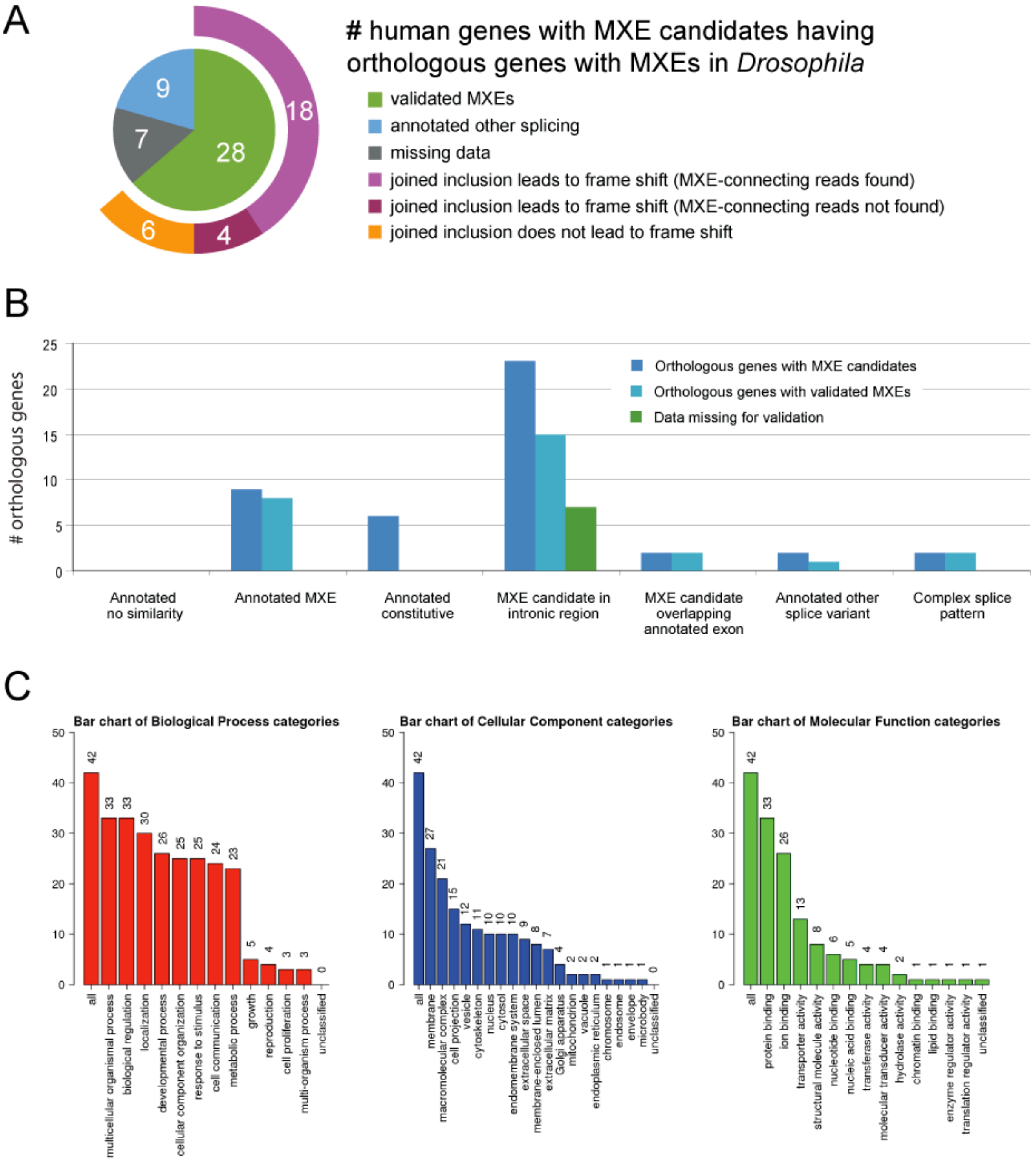

Appendix Fig. S34: Orthologous human and *Drosophila* genes containing MXEs. A) Orthologous genes in *Drosophila melanogaster* for all human genes containing MXE candidates were obtained with the Ensemble BioMart service. This list of orthologous genes was filtered with the list of *D.melanogaster* genes containing MXEs to obtain a list of genes with both types of exons, i) MXEs in human and MXEs in *D.melanogaster* (green slice), and ii) MXE candidates in human but validated to be spliced differently and MXEs in *D.melanogaster* (blue slice). Several of the human MXE candidates could not be validated because of missing SJ data (grey slice). Most of the validated MXE clusters in human contain exon-joining reads which would, however, lead to a frame-shift and premature stop codon in case of combined inclusion. These genes include the *SCN1A*, *SCN2A*, *SCN8A*, *SCN9A* sodium channels, the *GLRA2* receptor gene, and the *CACN1C* and *CACN1D* calcium channel genes. B) Distribution of the human MXE-candidate containing genes with orthologs in *Drosophila*

with respect to the annotation and novel prediction of the MXEs. C) GO enrichment analysis of the human MXE-containing genes with orthologs in *Drosophila*.

A

	human gene name	*Drosophila* gene name
homologous MXEs	ACTN2, ACTN4	actn
	GLRA2	GluClalpha
	KCNMA1	slo
	PNPLA6	sws
MXE in human and homologous exon in *Drosophila*	CACNA1A, CACNA1B	cac
	CEPT1	bbc
	KCNN3	SK
	SCN1A, SCN2A, SCN8A, SCN9A	para
	SNAP25	Snap25
	TCF7L2	pan
	TPM1, TPM2, TPM3	TM1
no exon similarity	CACNA1C, CACNA1D	Ca-alpha1D
	FAR1	CG30417
	LRP8	LpR1
	OBSCN	trol
	PNPLA6	sws
	SLIT2, SLIT3	sli

B **example for homologous MXEs**

ACTN2

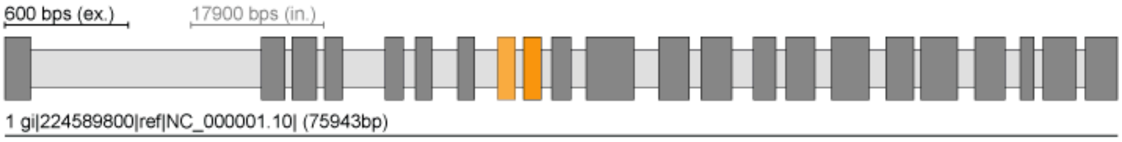

For clarity introns have been scaled down by a factor of 27.69

```
                      10        20
               ....|....|....|....|....|....
novel exon     DLVYTARPDERAIMTYVSCYYHAFAGAQK
annotated exon DIVNTPKPDERAIMTYVSCFYHAFAGAEQ
```

ACTN4

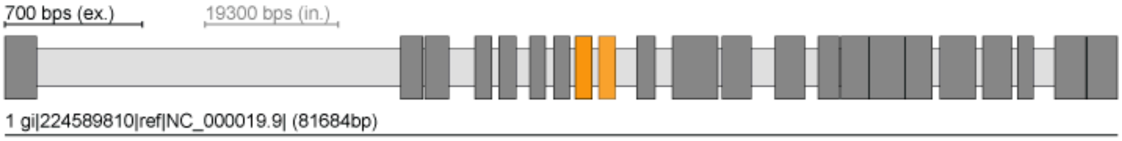

For clarity introns have been scaled down by a factor of 28.43

```
                      10        20
               ....|....|....|....|....|....
annotated exon DIVNTARPDEKAIMTYVSSFYHAFSGAQK
novel exon     DIVGTLRPDEKAIMTYVSCFYHAFSGAQK
```

*Dm*ACTN

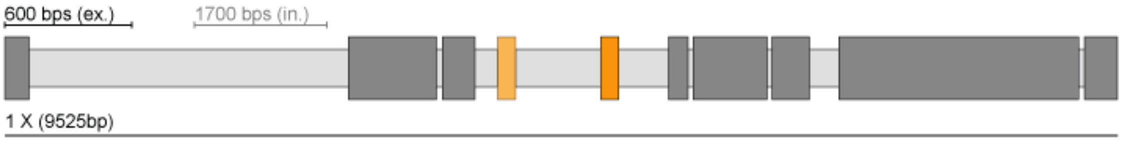

For clarity introns have been scaled down by a factor of 2.71

```
                      10        20
               ....|....|....|....|....|....
annotated MXE  DLINTPKPDERAIMTYVSCYYHAFQGAQQ
annotated MXE  DLQNTALPDERAVMTYVSSYYHCFSGAQK
```

Appendix Fig. S35: Orthologous human and *Drosophila* genes containing MXEs. A) The orthologous human and *Drosophila* genes containing MXEs were manually compared to determine whether the MXE clusters encode similar regions. The results were sorted by i) MXE clusters whose MXEs have identical exon phase, similar length and sequence similarity, and which code for the same region of the protein, ii) MXE clusters whose MXEs have an homologous exon in the respective other species with identical exon phase, similar length and sequence similarity, and which code for the same region of the protein, and iii) MXE clusters with no corresponding exons in the respective other species. B) The human α-actinin *ACTN2* and *ACTN4* genes are paralogs, and their *Drosophila* ortholog is *ACTN*. All three genes have an homologous cluster of two MXEs, the MXEs have identical exon phase, similar length and sequence similarity, and code for the same region of the protein. This homology could be explained by two scenarios: 1) the appearance of this MXE cluster predates the divergence of deuterostomes and protostomes, which would be an example of divergent evolution. 2) The clusters appeared independent of each other, which would be the result of convergent evolution.

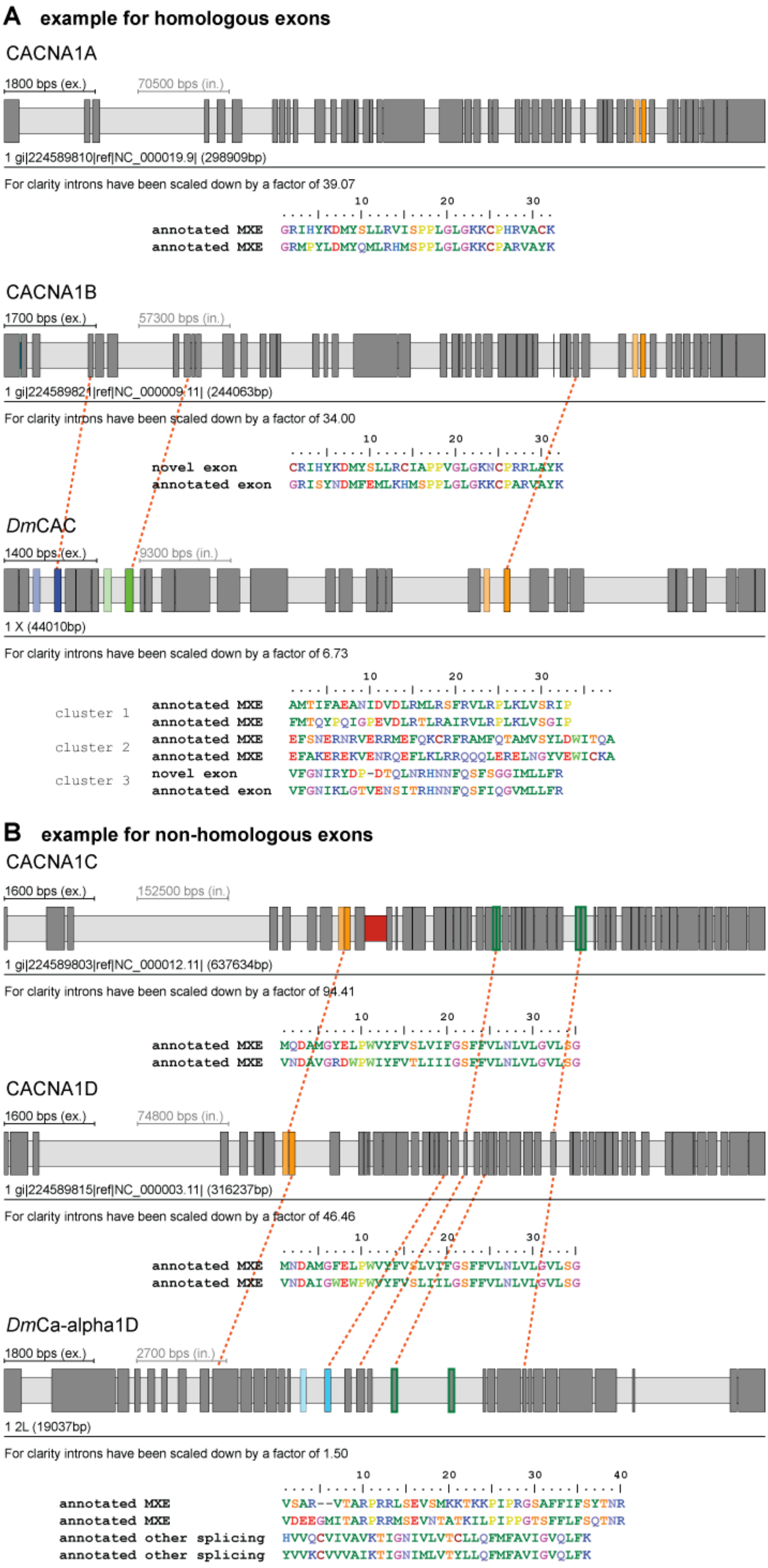
A example for homologous exons
CACNA1A
1800 bps (ex.)
70500 bps (in.)
1 gi|224589810|ref|NC_000019.9| (298909bp)
For clarity introns have been scaled down by a factor of 39.07
annotated MXE GRIHYKDMYSLLRVISPPLGLGKKCPHRVACK
annotated MXE GRMPYLDMYQMLRHMSPPLGLGKKCPARVAYK
CACNA1B
1700 bps (ex.)
57300 bps (in.)
1 gi|224589821|ref|NC_000009.11| (244063bp)
For clarity introns have been scaled down by a factor of 34.00
novel exon CRIHYKDMYSLLRCIAPPVGLGKNCPRRLAYK
annotated exon GRISYNDMFEMLKHMSPPLGLGKKCPARVAYK
DmCAC
1400 bps (ex.)
9300 bps (in.)
1 X (44010bp)
For clarity introns have been scaled down by a factor of 6.73
cluster 1
annotated MXE AMTIFAEANIDVDLRMLRSFRVLRPLKLVSRIP
annotated MXE FMTQYPQIGPEVDLRTLRAIRVLRPLKLVSGIP
cluster 2
annotated MXE EFSNERNRVERRMEFQKCRFRAMFQTAMVSYLDWITQA
annotated MXE EFAKEREKVENRQEFLKLRRQQQLERELNGYVEWICKA
cluster 3
novel exon VFGNIRYDP-DTQLNRHNNFQSFSGGIMLLFR
annotated exon VFGNIKLGTVENSITRHNNFQSFIQGVMLLFR
B example for non-homologous exons
CACNA1C
1600 bps (ex.)
152500 bps (in.)
1 gi|224589803|ref|NC_000012.11| (637634bp)
For clarity introns have been scaled down by a factor of 94.41
annotated MXE MQDAMGYELPWVYFVSLVIFGSFFVLNLVLGVLSG
annotated MXE VNDAVGRDWPWIYFVTLIIIGSFFVLNLVLGVLSG
CACNA1D
1600 bps (ex.)
74800 bps (in.)
1 gi|224589815|ref|NC_000003.11| (316237bp)
For clarity introns have been scaled down by a factor of 46.46
annotated MXE MNDAMGFELPWVYFVSLVIFGSFFVLNLVLGVLSG
annotated MXE VNDAIGWEWPWVYFVSLIILGSFFVLNLVLGVLSG
DmCa-alpha1D
1800 bps (ex.)
2700 bps (in.)
1 2L (19037bp)
For clarity introns have been scaled down by a factor of 1.50
annotated MXE VSAR--VTARPRRLSEVSMKKTKKPIPRGSAFFIFSYTNR
annotated MXE VDEEGMITARPRRMSEVNTATKILPIPPGTSFFLFSQTNR
annotated other splicing HVVQCVIVAVKTIGNIVLVTCLLQFMFAVIGVQLFK
annotated other splicing YVVKCVVVAIKTIGNIMLVTYLLQFMFAVIGVQLFK

Appendix Fig. S36: Examples of orthologous human and *Drosophila* genes containing MXEs. A) The human gene paralogs *CACNA1A* and *CACNA1B* are orthologs to the *Drosophila cac* gene. *CACNA1A* and *CACNA1B* both have a MXE cluster whose appearance predated their duplication. The *Drosophila cac* gene contains three clusters of MXEs. These three clusters have homologous exons in the human *CACNA1A* and *CACNA1B* genes (identical exon phase, similar length and sequence similarity, coding for the same region of the protein). B) The human gene paralogs *CACNA1C* and *CACNA1D* are orthologs to the *Drosophila Ca-alpha1D* gene. *CACNA1C* and *CACNA1D* contain a paralogous MXE cluster. The *Drosophila Ca-alpha1D* gene contains a single MXE cluster. In these gene orthologs, however, the exons encoding the MXEs are not homologous. The region encoding the MXE cluster in *CACNA1C* and *CACNA1D* is homologous to a region that is part of a larger exon in the *Drosophila Ca-alpha1D* gene. Similarly, the MXE-encoded part of the *Ca-alpha1D* gene is part of a larger exon in *CACNA1C* and *CACNA1D*. Clusters of MXEs are coloured. Exons, that resemble MXEs (neighbouring exons, identical exon phase, similar length and sequence) but were validated to be spliced differently, are indicated with dark green borders. Interestingly, these exons in *CACNA1C* and *Ca-alpha1D* encode parts of transmembrane regions. Thus, their complete absence or their combined inclusion (as indicated by their annotation as other splicing) would lead to a protein in which all subsequent parts are switched around the membrane: former outside parts would be inside, and former inside parts would be outside. From a protein structure view, these exons could only be spliced in a mutually exclusive manner. These exon annotations might be the result from mis-splicing events, where exon-joining reads were found which, however, do not lead to a frame-shift because of splice site incompatibility.
